# New Players in the Interaction Between Beetle Polygalacturonases and Plant Polygalacturonase-Inhibiting Proteins: Insights From Proteomics and Gene Expression Analyses

**DOI:** 10.3389/fpls.2021.660430

**Published:** 2021-06-04

**Authors:** Wiebke Haeger, Natalie Wielsch, Na Ra Shin, Steffi Gebauer-Jung, Yannick Pauchet, Roy Kirsch

**Affiliations:** ^1^Department of Entomology, Max Planck Institute for Chemical Ecology, Jena, Germany; ^2^Mass Spectrometry Research Group, Max Planck Institute for Chemical Ecology, Jena, Germany

**Keywords:** GH28, plant–insect interaction, herbivorous insect, *Phaedon cochleariae*, plant protection, *Brassica rapa* ssp. *pekinensis*, cell wall proteins, proteomics

## Abstract

Plants possess various defense strategies to counter attacks from microorganisms or herbivores. For example, plants reduce the cell-wall-macerating activity of pathogen- or insect-derived polygalacturonases (PGs) by expressing PG-inhibiting proteins (PGIPs). PGs and PGIPs belong to multi-gene families believed to have been shaped by an evolutionary arms race. The mustard leaf beetle *Phaedon cochleariae* expresses both active PGs and catalytically inactive PG pseudoenzymes. Previous studies demonstrated that (i) PGIPs target beetle PGs and (ii) the role of PG pseudoenzymes remains elusive, despite having been linked to the pectin degradation pathway. For further insight into the interaction between plant PGIPs and beetle PG family members, we combined affinity purification with proteomics and gene expression analyses, and identified novel inhibitors of beetle PGs from Chinese cabbage (*Brassica rapa* ssp. *pekinensis*). A beetle PG pseudoenzyme was not targeted by PGIPs, but instead interacted with PGIP-like proteins. Phylogenetic analysis revealed that PGIP-like proteins clustered apart from “classical” PGIPs but together with proteins, which have been involved in developmental processes. Our results indicate that PGIP-like proteins represent not only interesting novel PG inhibitor candidates in addition to “classical” PGIPs, but also fascinating new players in the arms race between herbivorous beetles and plant defenses.

## Introduction

As plants are restricted in their mobility, they have evolved sophisticated strategies to adapt to their constantly changing environment. Plants are challenged not only by abiotic stresses but also by phytopathogenic microorganisms and herbivores, all of which exploit the plants as sources of nutrients. Thus, plant defense is a complex, multifaceted system in which physical barriers, chemical compounds and defense proteins intertwine and adapt to counter these vastly different attackers. Chemical defenses are as versatile as the secondary metabolites involved, which can be repellent, antinutritional or toxic, and may even attract natural enemies of herbivores (Croteau et al., 2000; Mithöfer and Boland, 2012). Similarly, proteinaceous defenses also act on multifarious fronts. For example, they contribute to strengthening the mechanical barriers through protein agglutination or cross-linking to cell wall components (Rashid, 2016). Harmful proteins or enzymes can directly target structures in pathogens or herbivores (Edreva, 2005). For instance, chitinases possess antifungal activity by cleaving fungal cell wall components (Kumar et al., 2018), and lectins impair carbohydrate structures in the insect gut, which has detrimental consequences for the herbivore (Vandenborre et al., 2011). Furthermore, enzymes produced by pathogens or herbivores can be inhibited in the apoplast or the insect gut. For example, the overexpression of protease inhibitors impaired fungal growth (Yarullina et al., 2016) and reduced the survival of beetles (Delledonne et al., 2001). Plants overexpressing inhibitors targeting α-amylases were more resistant than the wild-type plants and drastically decreased the survival of several beetle species (Kaur et al., 2014). Enzymes macerating the plant cell wall are versatile, and inhibiting them enhanced plant resistance to various pathogens (Kalunke et al., 2015; Ma et al., 2017).

A fascinating example of such enzyme inhibitors are polygalacturonase-inhibiting proteins (PGIPs). These extracellular leucine-rich repeat (LRR) proteins are ubiquitously distributed in plants (Kalunke et al., 2015). Localized in the plant cell wall (Spadoni et al., 2006; Protsenko et al., 2008), they bind and inhibit polygalacturonases (PGs, EC 3.2.1.15) of the glycoside hydrolase family 28 (GH28) (De Lorenzo et al., 2001). PGs hydrolyze pectin, a galacturonic acid-rich polysaccharide of the plant cell wall (Mohnen, 2008). The inhibition of PG activity by PGIPs restricts tissue maceration and favors the formation of oligogalacturonides, which elicit plant defenses (De Lorenzo et al., 1994; De Lorenzo and Ferrari, 2002). Additionally, PGIPs can protect pectin by binding to and thus shielding the substrate from degradation (Spadoni et al., 2006; Protsenko et al., 2008). Some PGIPs are constitutively expressed, whereas others are induced in response to various environmental cues such as wounding, the presence of oligogalacturonides, pathogen infection or herbivory (Li et al., 2003; D’Ovidio et al., 2004b; Hwang et al., 2010; Kirsch et al., 2020). PGIPs have been studied best and extensively reviewed in the context of plant-microbe interactions (De Lorenzo et al., 2001; Kalunke et al., 2015; Rathinam et al., 2020). PGs, secreted into the apoplast upon infection, often represent pathogenicity factors (Isshiki et al., 2001; Oeser et al., 2002; Bravo Ruiz et al., 2016). Hence, reduced PGIP levels in plants led to increased susceptibility (Ferrari et al., 2006; Hou et al., 2015), whereas overexpression of PGIPs contributed to plant resistance to phytopathogens and also reduced disease symptoms (Powell et al., 2000; Ferrari et al., 2003; Manfredini et al., 2005).

Besides their abundance in microbes, PGs are also widespread in insects and have been described for numerous orders (Calderón-Cortés et al., 2012; Shelomi et al., 2016; McKenna et al., 2019) including Hemiptera (mirid bugs) (Frati et al., 2006; Allen and Mertens, 2008) and Coleoptera (beetles, mainly of the “Phytophaga” clade (Pauchet et al., 2010; Kirsch et al., 2014, 2016). Initially, PGIPs were shown to reduce insect PG activity (Doostdar et al., 1997; D’Ovidio et al., 2004b; Frati et al., 2006) and the resistance of mung bean (*Vigna radiata*) to seed beetles (*Callosobruchus* spp.) was associated with genes encoding putative PGIPs (Chotechung et al., 2016; Kaewwongwal et al., 2020). We recently found BrPGIP3 from Chinese cabbage (*Brassica rapa* ssp. *pekinensis*) to be a potent inhibitor of individual PGs from the mustard leaf beetle *Phaedon cochleariae* as well as of gut PG activity *in vitro* (Haeger et al., 2020). Moreover, *P. cochleariae* larvae were negatively influenced *in vivo* by the presence of PGIPs in different *Arabidopsis thaliana* lines (Kirsch et al., 2020).

Polygalacturonases – like their antagonist, PGIPs – often belong to large multi-gene families, which have been shaped by an evolutionary arms race (Casasoli et al., 2009). In the PG-PGIP interaction, specificity is maintained by a few positively selected amino acid hotspots (Casasoli et al., 2009; Rathinam et al., 2020). Exchanging a single amino acid in the PG or PGIP may alter their reciprocal recognition, allowing proteins to bind novel partners or an existing interaction to be circumvented (Leckie et al., 1999; Benedetti et al., 2013; Chen et al., 2019). Thus, expanding the number of *PG* genes may facilitate a subfunctionalization to achieve a more effective pectin degradation, and also be a strategy to escape inhibition by PGIPs. Likewise, PGIPs are highly variable in their LRR domain, which is typically involved in protein-protein interactions (Kobe and Kajava, 2001). Such variability has allowed them to flexibly co-evolve with PGs (Casasoli et al., 2009), bind to new ligands and acquire novel functions (Meyer et al., 1999). In *P. cochleariae*, nine PG family members have been identified in the beetle gut (Kirsch et al., 2012, 2014); among these, PCO_GH28-1 and other endo-PGs catalyze the random hydrolysis of homogalacturonan, the main component of pectin (Kirsch et al., 2014). Interestingly, some PG family members, including PCO_GH28-3, were inactive on all tested substrates and possessed amino acid substitutions in residues involved in catalysis or substrate binding (Kirsch et al., 2012, 2014). Nonetheless, recent findings suggest that these pseudoenzymes play a role in the pectin digestion pathway, because when they were silenced, food-to-energy conversion in *P. cochleariae* larvae was less efficient compared to when pseudoenzymes were not silenced (Kirsch et al., 2019). Even though such pseudoenzymes have also been described in other beetles (Kirsch et al., 2014, 2016; Pauchet et al., 2014), their function remains elusive.

In this study, we combined affinity purification with proteomics and gene expression analysis to identify several BrPGIPs from *B. rapa* ssp. *pekinensis* as novel putative inhibitors of the beetle PG PCO_GH28-1. A comparison of interacting proteins showed, however, that the pseudoenzyme PCO_GH28-3 was not targeted by any BrPGIPs. Surprisingly, two BrPGIP-like proteins interacted with both tested PG family members, and one of the proteins was induced by herbivory. Phylogenetic analysis revealed that these BrPGIP-like proteins evolved independently from “classical” PGIPs in the Brassicaceae and clustered together with proteins, which so far have been only linked to developmental processes. BrPGIP-like proteins have not previously been associated with the pectin degradation pathway, an association that makes them interesting new candidates for PG inhibitors.

## Materials and Methods

### Plants and Beetles

Chinese cabbage (*B. rapa* ssp. *pekinensis* “Cantonner Witkrop”) was reared in the greenhouse under long day conditions (21°C, 55% humidity, 14 h/10 h light/dark period). Mustard leaf beetle *P. cochleariae* larvae and adults were kept on *B. rapa* ssp. *pekinensis* leaves as a continuous culture (20°C, 16 h/8 h light/dark period).

### Expression of PCO_GH28-1 and -3 in *Pichia pastoris* and Purification

The open reading frames (ORFs) of PCO_GH28-1 and -3 (HE962193.1 and HE962195.1) were cloned into pIB/V5-His-TOPO^®^ TA (Thermo Fisher Scientific, Waltham, MA, United States) to attach a C-terminal His_6_ and V5 epitope as described previously (Kirsch et al., 2014). Subsequently, the native signal peptides (identification by SignalP 4.1; Petersen et al., 2011) were replaced by the vector’s secretion signal peptide during the cloning into the yeast expression vector pPICZα A (Thermo Fisher Scientific). The primers (Supplementary Table 1) were designed in such a way that they introduced recognition sequences for the restriction enzyme *Pml*I upstream of the PG ORFs. A recognition site for *Kpn*I as well as a translation termination signal was introduced downstream of the ORFs, including the V5 and the His6 epitope from the pIB/V5-His-TOPO^®^ vector into the amplified sequence. The constructs were verified by sequencing. The PG family members were expressed in *Pichia pastoris* following the manufacturer’s instructions of the EasySelect^TM^ Pichia Expression Kit (Thermo Fisher Scientific) with slight modifications. Instead of a single colony, a dense buffered glycerol-complex medium (BMGY) culture was used to inoculate a BMGY pre-culture to be able to accurately calculate the growth, as a precise OD_600_ turned out to be essential for expression success. Protein expression was induced with 1% methanol either approximately every 12 h or in alternating intervals of 8 and 16 h, keeping one treatment constant for one expression. Expression was monitored by western blot using an anti-V5-HRP antibody (#R961-25, Invitrogen, Thermo Fisher Scientific), diluted 1:20 000 in 5% milk powder in Tris-buffered saline with 0.1% Tween20 (TBST).

The expression culture medium was dialyzed overnight in SnakeSkin^TM^ Dialysis Tubing (35 mm I.D., 10K MWCO, Thermo Fisher Scientific) against immobilized metal ion chromatography (IMAC) binding buffer (50 mM sodium phosphate buffer pH 7.4, 0.5 M NaCl). Potential precipitate occurring during dialysis was removed by centrifugation (10000 × *g*, 5 min). The His_6_-tagged proteins were purified by IMAC using HiTrapTM^TM^ TALON cobalt agarose resin in either a batch or fast protein liquid chromatography (FPLC). For batch purification, the dialyzed protein samples were incubated rotating with HiTrap^TM^ TALON Superflow agarose beads (GE Healthcare Life Sciences, München, Germany) for 1 h at 4°C. Afterward the sample was poured onto a gravity flow column retaining the beads and washed with at least 10 column volumes (CV) of IMAC wash buffer (50 mM sodium phosphate buffer pH 7.4, 0.3 M NaCl, 10 mM imidazole). Elution was achieved in a multi-step process. 1 CV of IMAC elution buffer (50 mM sodium phosphate buffer pH 7.4, 0.3 M imidazole) was applied onto the column. The displaced liquid still containing mainly wash fraction was collected separately (E0). After capping the bottom of the column, an additional 1 CV of IMAC elution buffer was added. The column was incubated for 5 min, and the elution drained from the column by gravity was collected as E1. This was repeated three times. FPLC purification was carried out with an ÄKTA FPLC Protein Purification System (GE Healthcare Life Sciences) and pre-packed HiTrap^TM^ TALON crude 1 ml columns (Merck KGaA, Darmstadt, Germany). After sample loading, the column was washed with 10 CV IMAC wash buffer. Elution was performed in a stepwise manner as described for batch purification by pausing and resuming the FPLC ÄKTA System manually for every 1 CV of IMAC elution buffer. Samples were taken at every purification step to monitor the success and efficiency of the protein purification by SDS-PAGE (Supplementary Figure 1A) and western blot. As PCO_GH28-1 has been observed to fragment with increasing temperatures, additional bands in the western blot were analyzed by LC-MS/MS to verify purification (Supplementary Figure 1B and Supplementary Table 2).

### Cross-Linking of Pectin and Interaction Assay With Insoluble Pectin

To test the binding of the PG family members to pectic substrates, 20 μl of PG-containing culture medium was incubated with 50 μl of either cross-linked pectin (CLP) or methylated CLP (mCLP) (50% slurry) and 30 μl H_2_O at 4°C under constant inversion. Substrates were prepared as described earlier (Kirsch et al., 2016). The insoluble CLP and mCLP were separated from the supernatant by centrifugation (2 min, 16000 × *g*, 4°C). The pellet was washed thrice with 50 mM citrate phosphate buffer pH 5.0, keeping the first 500 μl as wash fraction and discarding the other 2 ml. After resuspension of the pellet in 100 μl H_2_O, culture medium, supernatant, wash and pellet fraction were applied onto a western blot to monitor the PG localization. Volumes of supernatant, wash and pellet were adjusted in such a way that they were equivalent to the initially used amount of the culture medium.

### Pull-Down Assay

#### Immobilization of PG Family Members on Column

Purified protein samples were pooled and concentrated with Pierce^TM^ Protein Concentrators 9K MWCO, 20 mL and transferred into AL coupling buffer BupH^TM^ Phosphate Buffered Saline pH 7.2 with Zeba^TM^ Spin Desalting Columns according to the manufacturers’ instructions (all from Thermo Fisher Scientific). Protein concentrations were determined by Quick Start^TM^ Bradford Protein Assay (Bio-Rad Laboratories GmbH, Feldkirchen, Germany). 1.73 and 0.92 mg of PCO_GH28-1 and -3, respectively, were immobilized on a column using the AminoLink^®^ Plus Immobilization Kit (Thermo Fisher Scientific) overnight at 4°C, according to the manufacturer’s instructions. Each flow-through of the column preparation was saved to monitor protein coupling efficiency and potential loss of protein from the column. The columns were stored upright at 4°C until further use. To ensure that the treatment and storage did not impair protein function, PG activity was verified for AminoLink^®^ agarose-coupled PCO_GH28-1 by thin layer chromatography [TLC; as described previously (Kirsch et al., 2014)] using demethylated polygalacturonic acid (PGA; 1% w/v in H_2_O, P-PGACT, Megazyme Ltd., Bray, Ireland).

#### Isolation of Cell Wall Proteins (CWPs)

Cell wall proteins were isolated from 6-week-old *B. rapa* ssp. *pekinensis* plants that had previously been challenged with herbivores (15 adult and 30 larval *P. cochleariae* per plant for 3 days) in a protocol adapted from Feiz et al. (2006). Cell walls were isolated from above-ground parts of the plants by homogenization and step-wise centrifugation in increasing sucrose concentrations (5 mM sodium acetate with 0.2 – 1 M), washed with pre-cooled 5 mM sodium acetate buffer pH 4.6 on Miracloth filtration material (pore size 22 – 25 μm, Merck KGaA), ground in liquid nitrogen and lyophilized. Sucrose gradients were used to limit the contamination of the wall fraction with intracellular proteins, as organelles and other vesicles are less dense than cell wall polysaccharides. Proteins were extracted from the cell walls with increasing salt concentrations. Two extractions were performed with 25 – 35 ml of protein extraction solutions [5 mM sodium acetate containing 0.2 M CaCl_2_ or 1 M NaCl (instead of 2 M LiCl)], respectively. The supernatants of each respective extraction were united and dialyzed against ultrapure water overnight at 4°C in SnakeSkin^TM^ Dialysis Tubing (35 mm I.D., 10K MWCO, Thermo Fisher Scientific). The CWP extracts were lyophilized and resuspended in AL binding/wash buffer (50 mM citrate phosphate buffer pH 5.0, 0.15 M NaCl). Protein concentrations were determined by Quick Start^TM^ Bradford Protein Assay (Bio-Rad Laboratories GmbH).

#### Affinity Chromatography

To remove proteins that bind unspecifically to the column, 4.3 mg of CWPs were run over AminoLink^®^ resin (pre-treatment), that had been treated with water instead of protein sample (empty column). The interaction assay between *B. rapa* ssp. *pekinensis* CWPs and the immobilized PG family members on the columns was performed following the manufacturer’s instructions of AminoLink^®^ Plus Immobilization Kit (Thermo Fisher Scientific). Each half of the pre-treated CWPs was incubated with the PCO_GH28-1 and -3 columns, respectively. After gravity-driven draining of the flow-through, the column was washed with 6 CV of AL binding/wash buffer. Elution was achieved in a stepwise manner. One CV of AL elution buffer (0.1 M Glycine HCl pH 2.0) was added onto the column and the flow-through, which was still part of the washing fraction, was collected as E0. Then the column was capped and incubated with another 1 CV of AL elution buffer for 5 min. The elution was collected as E1, and this procedure was repeated three times. Ten μl of AL neutralization buffer (1 M Tris) was added to 2 ml of elution. The columns were regenerated by washing with 8 CV of AL binding/wash buffer and equilibrated with 4 CV of storage buffer (AL coupling buffer, 0.05% sodium azide) and stored at 4°C.

Additionally, CWPs were analogously applied onto an empty column as control. The elutions were treated like the ones from the GH28 columns to distinguish between proteins that bound only to the column material and those that interacted specifically with PCO_GH28-1 and -3.

#### TCA Precipitation and SDS-PAGE

The eluted proteins were precipitated with trichloroacetic acid (TCA) to enrich them for subsequent analyses. Sodium deoxycholate was added to a final concentration of 0.02%. Samples were mixed, placed on ice and supplemented with TCA to a final concentration of 10%, and then incubated on ice for 1 h. Precipitated proteins were pelleted by centrifugation (10 min, 16000 × *g*, 4°C), washed with 100% ice-cold acetone by incubating them on ice for 15 min and subsequent centrifugation (10 min, 16000 × *g*, 4°C). Washing was repeated once; the pellet was air-dried and boiled in SDS-PAGE buffer (7.5 μl 4x XT Sample Buffer, 1.5 μl 20x XT Reducing Agent, 1% SDS, ad 30 μl H2O) for 5 min at 95°C. The samples were separated on Criterion XT Bis-Tris Precast Gels (125 V for 1.5 h in XT MES Running Buffer) (all from Bio-Rad Laboratories GmbH). PageRuler Plus Prestained Protein Ladder (Thermo Fisher Scientific) was used as a size standard. For a subsequent Coomassie staining, the gel was equilibrated in H_2_O for 5 min, incubated in 100 ml acetic acid/ethanol/H_2_O (10:40:50) for 1 h and subsequently rehydrated with H_2_O (three times, 10 min). The gel was stained with PageBlue^TM^ Protein Staining Solution (Thermo Fisher Scientific) overnight and repeatedly washed with H_2_O for destaining.

### NanoLC-MS/MS Analysis and Protein Identification

#### In-Gel Digestion of Proteins

Protein bands of interest were cut out from Coomassie-stained gels, cut into small pieces, washed several times with 25 mM NH_4_HCO_3_ and destained with 50% acetonitrile (ACN)/25 mM NH_4_HCO_3_. The proteins were then reduced with 10 mM DTT at 50°C for 1 h and alkylated with 55 mM iodoacetamide (IAA) at room temperature (RT) in the dark for 45 min. Next, destained, washed, dehydrated gel pieces were rehydrated for 1 h in 12 ng/μl solution of porcine trypsin (Promega GmbH, Mannheim, Germany) in 25 mM NH_4_HCO_3_ at 4°C and incubated overnight at 37°C. The tryptic peptides were extracted from gel pieces with 75% ACN/5% formic acid (FA), and dried in a SpeedVac (Shevchenko et al., 2007). For nanoUPLC-MS/MS, analysis samples were reconstructed in 10 μl aqueous 1% FA.

#### NanoLC-MS/MS Analysis

Each sample was injected onto a nanoAcquity nanoUPLC system online coupled to a Q-ToF HDMS mass spectrometer (both Waters, Manchester, United Kingdom). Peptides were initially transferred with 0.1% aqueous FA for desalting onto a Symmetry C18 trap-column (20 × 0.18 mm, 5 μm particle size) at a flow rate of 15 μl/min (0.1% aqueous FA), and subsequently eluted onto a nanoAcquity C18 analytical column (200 mm × 75 μm ID, BEH 130 material, 1.7 μm particle size) at a flow rate of 350 nl/min with the following gradient: 1 – 30% B over 13 min, 30 – 50% B over 5 min, 50 – 95% B over 5 min, isocratic at 95% B for 4 min, and a return to 1% B over 1 min (phases A and B were composed of 0.1%FA and 100% ACN in 0.1% FA, respectively). The analytical column was re-equilibrated for 9 min prior to the next injection.

The eluted peptides were transferred to the nanoelectrospray source of a Synapt HDMS tandem mass spectrometer (Waters) that was operated in V-mode with a resolution power of at least 10000 FWHM. All analyses were performed in positive ESI mode. A 650 fmol/μl human Glu-fibrinopeptide B in 0.1% FA/ACN (1:1 v/v) was infused at a flow rate of 0.5 μl/min through the reference Nano-LockSpray source every 30 s to compensate for mass shifts in MS and MS/MS fragmentation mode. LC-MS data were collected using data-dependent acquisition (DDA). The acquisition cycle consisted of a survey scan covering the range of m/z 400 – 1500 Da followed by MS/MS fragmentation of the four most intense precursor ions collected at 1 s intervals in the range of 50 – 2000 m/z. Dynamic exclusion was applied to minimize multiple fragmentations for the same precursor ions.

#### Data Processing and Protein Identification

Data-dependent acquisition raw files were collected using MassLynx v4.1 software and processed using ProteinLynx Global Server Browser (PLGS) v2.5 software (Waters) and pkl-files were generated. Using Mascot software version 2.6.0 (Matrix Science Inc., London, United Kingdom), pkl-files of MS/MS spectra were searched against the NCBInr database (updated May 24, 2020). The following searching parameters were applied: fixed precursor ion mass tolerance of 15 ppm for survey peptide, fragment ion mass tolerance of 0.1 Da, one missed cleavage, fixed carbamidomethylation of cysteines and possible oxidation of methionine. Hits were considered confident if at least one peptide was matched in the Mascot analysis with an ion score that indicated identity or extensive homology (*p* < 0.05). If multiple hits were detected for a certain peptide combination, the hit that originated from *B. rapa* ssp. *pekinensis* or the plant most closely related to *B. rapa* ssp. *pekinensis* was selected. From multiple hits of the same plant and if possible, we selected annotated hits over uncharacterized, predicted or partial sequences. The mass spectrometry proteomics data have been deposited to the ProteomeXchange Consortium via the PRIDE (Perez-Riverol et al., 2019) partner repository with the dataset identifier PXD022562 and doi: 10.6019/PXD022562.

#### Bioinformatic Analysis

The protein sequences of the confident hits were screened for the presence of a signal peptide for extracellular secretion using SignalP 5.0 (Petersen et al., 2011) and DeepLoc 1.0 (Almagro Armenteros et al., 2017). Proteins were considered as CWPs, if both methods predicted the presence of a signal peptide for the secretory pathway as well as an extracellular localization. CWPs were considered to be LRR proteins if they possessed at least one predicted LRR domain based on LRRfinder^1^ (upper and lower boundaries at 95 and 80%, respectively) and were predicted to contain “Leucine-rich-repeat domains (LRRs)” according to InterProScan^2^. Based on their GO terms, the InterProScan, a BLAST search^3^ and a subsequent annotation of an EC number, CWPs were grouped into nine categories proposed by Jamet et al. (2008).

#### Identification of PGIPs and PGIP-like Proteins

BrPGIP1 to -9 and BrPGIP-like1 to -like5 were initially identified by BLAST searches against the *B. rapa* ssp. *pekinensis* genome and proteome (B. rapa v2.5) in the *Brassica* database (BRAD^4^) (Wang et al., 2011; Cai et al., 2017) using *Brassica napus* PGIPs as queries. As the genome was generated from a different cultivar (“Chiifu-401-42”) than the one used in our study (“Cantonner Witkrop”), full-length sequences of all PGIPs were confirmed by PCR, and subsequent sequencing using an RNA pool isolated from leaves of the latter cultivar. RNA isolation and cDNA synthesis were performed as described below. The full-length coding sequence of *BrPGIP7* (accession MW264493) was confirmed for the first time as the corresponding gene was fragmented and not complete in the v2.5 assembly. The presence of another newly detected BrPGIP1.1 (accession MW264492), which we named based on its high similarity to BrPGIP1 (three amino acid deletions and four amino acid exchanges), was confirmed by PCR and sequencing as well as by discriminative peptides in the LC-MS/MS Mascot analysis.

### Phylogeny of PGIPs

PGIP family members used for the phylogenetic analysis were extracted following BLAST searches against the NCBInr database using multiple characterized PGIPs as queries. Alignment of 97 PGIP amino acid sequences and 7 outgroup sequences (Supplementary Table 3) was performed with the MAFFT (v.1.3.7) algorithm (Supplementary Figure 2). We inferred maximum likelihood analyses using IQtree^5^, and ultrabootstrap support values were calculated with 1000 replications. The best fits model automatically determined by IQtree is JTTDCMut + I + G4.

### Effect of Herbivore Feeding and Wounding

#### Plant Treatment

Five-week-old *B. rapa* ssp. *pekinensis* plants were moved to a climate chamber 2 days before the induction experiment to adapt to the conditions (21°C, 50% humidity,10 h/14 h light/dark period). Plants were exposed to the following stresses: Five adults and ten larvae of *P. cochleariae* were placed on each leaf, and the plants were wounded in approximately 3 cm intervals with sterile serrated forceps at *t* = 0, 8, and 24 h to simulate the mechanical part of herbivore feeding. Untreated plants were used as controls. Three leaves per plant were treated and isolated, that is, covered with cellophane bags closed around the leaf stalks with sponge cloth and rubber bands. After 26 h, an approximately 10 cm diameter piece was cut from the leaf (avoiding the mid-rib if possible) and immediately frozen in liquid nitrogen.

#### RNA Isolation and Purification

Total RNA was isolated from approximately 100 mg of plant tissue ground in liquid nitrogen. The material was further homogenized in 1 ml Trizol with metal beads at 50 Hz for 5 min in a Tissue Lyzer (Qiagen GmbH, Hilden, Germany). All centrifugations were carried out at 12000 × *g* and 4°C. After 10 min of centrifugation, the supernatant was mixed with 200 μl chloroform, incubated for 3 min at RT. Centrifugation for 15 min separated phases with the RNA remaining in the upper aqueous phase. After transfer of the upper phase to a new tube, the RNA was pelleted by incubation with 500 μl isopropanol for 10 min at RT and subsequently centrifuged for 10 min. The RNA was washed with 75% ethanol once and pelleted by centrifugation (5 min, 7500 × *g*, 4°C). The pellet was air-dried for approximately 10 min, then dissolved in 89 μl RNase-free water. Afterward, the RNA was treated with 1 μl of TURBO DNase (Thermo Fisher Scientific) with 10 μl of 10X TURBO DNase Buffer for 30 min at 37°C. Subsequent purification was carried out with the RNeasy MinElute Cleanup Kit (Qiagen GmbH) according to the manufacturer’s instructions. The RNA concentration was quantified with a NanoDrop ND-1000 Spectrophotometer (Eppendorf AG, Hamburg, Germany). RNA integrity and quality were monitored using the RNA 6000 Nano Kit on a 2100 Bioanalyzer System (both Agilent Technologies, Inc., Santa Clara, CA, United States) according to the manufacturer’s instructions.

#### cDNA Synthesis

The cDNA synthesis was performed with the Verso cDNA Synthesis Kit (Thermo Fisher Scientific) using a modified version of the manufacturer’s instructions. 1 μg of template RNA was pre-incubated with 1 μl of RNA Primer (3:1 Random Hexamer:Anchored Oligo dT) in a total volume of 12 μl for 5 min at 70°C. After adding the remaining components on ice, reverse transcription was performed at 42°C for 60 min, followed by 30 min at 50°C. The cDNA volume was adjusted to 80 μl and stored at –20°C.

#### Real-Time qPCR

Real-time qPCR was performed in optical 96-well plates on a CFX machine (both Bio-Rad Laboratories GmbH) using ABsolute Blue qPCR SYBR Green Mix Plus ROX Vial (Thermo Fisher Scientific) according to the manufacturer’s instructions. Primers were designed using Primer3 (Koressaar and Remm, 2007; Untergasser et al., 2012; Koressaar et al., 2018; Supplementary Table 1). Our primers for *BrPGIP1* do not differentiate between *BrPGIP1* and *BrPGIP1.1*. Primer efficiency was calculated from template dilution series, and specific transcript amplification was verified by melting curve analysis. Transcripts were amplified from 1 μl of cDNA (*n* = 5), and the Cq threshold was automatically determined by the CFX Manager^TM^ Software (Bio-Rad Laboratories GmbH). *UBIQUITIN-CONJUGATING ENZYME 21* (*UBC21*, Bra009857) was used as a reference gene to quantify the expression of genes of interest. Statistical analysis was performed with SigmaPlot (Systat Software, San Jose, CA, United States) with an ANOVA (for normally distributed samples with equal variances) or ANOVA on Ranks (Kruskal–Wallis, for samples that were not normally distributed or had unequal variances) and a subsequent *post hoc* analysis (Student–Newman–Keuls).

### Cross-Linking Interaction Assay

#### Expression of BrPGIP-like1 and PCO_GH28s

BrPGIP-like1 (accession XP_009120892) was cloned into pPICZα A from *B. rapa* ssp. *pekinensis* cDNA. The primers (Supplementary Table 1) were designed in such a way that the native signal peptide (identification by SignalP 4.1; Petersen et al., 2011) was replaced by the vector’s secretion signal peptide. The recombinant protein included the His_6_ and myc epitope from the expression vector. PCO_GH28-1 to -9 were cloned into pIB/V5-His-TOPO^®^ TA as described previously (Kirsch et al., 2014). All constructs were verified by sequencing. BrPGIP-like1 was expressed in *P. pastoris* as described above, while PCO_GH28-1, -2, -3, -4, -5, -6, -8, and -9 were transiently expressed in Sf9 insect cells in 6-well plates (two wells per construct) as described previously (Kirsch et al., 2014). PCO_GH28-7 could not be expressed. Protein abundance was monitored by western blot using antibodies against the myc (BrPGIP-like1) or the V5 (PCO_GH28s) epitopes.

### Cross-Linking and Western Blot

We tested the interaction of PG family members with PGIPs in a cross-linking assay modified from Benedetti et al. (2011) and Haeger et al. (2020). It was essential to use the BrPGIP-like1 culture medium immediately on the day of harvest to avoid the formation of aggregates that occurs upon storage. Twenty-one μl of culture medium from BrPGIP-like1 expression was co-incubated with 5 μl of PCO_GH28 culture medium for 1 h at 4°C with 8 μl 0.2 M citrate phosphate buffer pH 5.0. Subsequently, 11.33 μl formaldehyde [4% in 1x phosphate-buffered saline (PBS), final concentration 1%] or 1x PBS was added and incubated overnight at 16°C. Single protein controls were incubated with culture medium from wild-type cells. Equal volumes of samples were applied onto an SDS-PAGE gel without boiling the sample to avoid a heat-induced reversal of formaldehyde cross-linking (Klockenbusch and Kast, 2010). SDS-PAGE was performed as described above, and proteins were subsequently blotted onto an Immun Blot PVDF Membrane (Bio-Rad Laboratories GmbH) at 100 V for 30 min. The membrane was blocked with 5% milk powder in TBST at RT for 1 h and incubated overnight with an anti-myc-HRP (A190-104P, Bethyl Laboratories, Inc., Montgomery, TX, United States) or anti-V5-HRP antibody (#R961-25, Invitrogen, Thermo Fisher Scientific), diluted 1: 300 000 and 1:20 000 in 5% milk powder in TBST, respectively. BrPGIP-like1 and the PCO_GH28s can be distinguished by their respective tags. Their chemiluminescence with SuperSignal^TM^ West Dura Extended Duration Substrate Kit (Thermo Fisher Scientific) was documented using Amersham Hyperfilm DCL chemiluminescence films (GE Healthcare Life Sciences), GBX Developer and Replenisher and GBX Fixer and Replenisher (both Kodak GmbH, Stuttgart, Germany).

## Results

### Interaction of *B. rapa* ssp. *pekinensis* CWPs With PCO_GH28-1 and -3

In an unbiased interaction assay, we aimed to first, identify new putative PG inhibitors for beetle PGs and second, test if catalytically inactive pseudoenzymes are still targeted by PGIPs. Therefore, we isolated CWPs from *B. rapa* ssp. *pekinensis* and, using LC-MS/MS and bioinformatic tools, confirmed that this cell wall proteome correlated with proteomes from previous studies (Ligat et al., 2011; Albenne et al., 2013; Calderan-Rodrigues et al., 2019), making it suitable starting material for our interaction assay (Supplementary Table 4 and Supplementary Figure 3). We chose PCO_GH28-1 and -3 as representatives of an active endo-PG and a catalytically inactive pseudoenzyme, respectively. In addition to previous studies in which PCO_GH28-3 did not hydrolyze any of the substrates tested (Kirsch et al., 2012, 2014, 2019), we showed that unlike PCO_GH28-1, the pseudoenzyme no longer binds to pectic substrates (Supplementary Figure 4).

**FIGURE 1:**
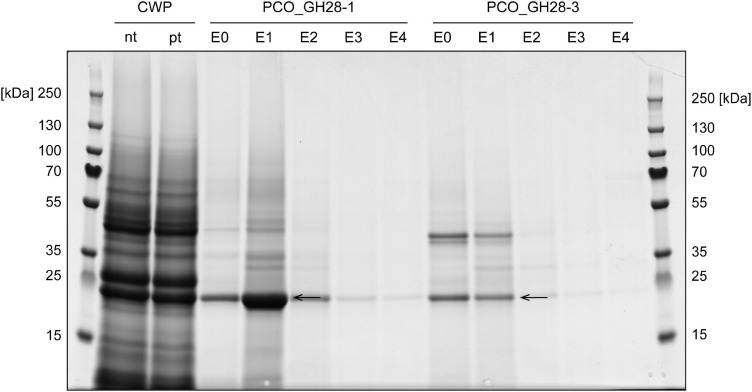
SDS-PAGE of pull-down assay of PCO_GH28-1 and -3 with *B. rapa* ssp. *pekinensis* cell wall proteins (CWPs). Non-treated (nt) CWP extracts were pre-treated (pt) with column resin to reduce unspecific binding to the PCO_GH28 columns and then passed over columns with immobilized purified PCO_GH28-1 and -3. Elution fractions E1 were analyzed by LC-MS/MS. A protein band detected in the negative control as well (Supplementary Figure 5) is indicated by the arrows. For detailed information on interacting LRR and total proteins, see Tables 1, 2 as well as Supplementary Tables 4–6. E0–4: elution fractions 1–4.

We tested the interaction of *B. rapa* ssp. *pekinensis* CWPs with the two *P. cochleariae* PG family members. Proteins interacting with either PCO_GH28-1 or -3 were separated by SDS-PAGE and analyzed by LC-MS/MS. The elution fractions from the PCO_GH28-1 and -3 columns resulted in two distinct protein band patterns (Figure 1). To narrow down promising candidates, we focused on extracellular LRR proteins from Brassicaceae plants (Tables 1, 2). A full list of interacting proteins can be found in Supplementary Tables 5, 6 (PCO_GH28-1 and -3) as well as Supplementary Table 7 (negative control). Proteins in the conspicuous band marked with an arrow in Figure 1 (PCO_GH28-1 and -3) and Supplementary Figure 5 (negative control) bound unspecifically to the column material and did not contain any LRR proteins. Thus, the interaction of BrPGIPs and BrPGIP-like proteins with the PG family members in our pull-down assay was specific.

**TABLE 1:** Extracellular LRR protein hits from a pull-down assay of PCO_GH28-1 with *B. rapa* ssp. *pekinensis* CWPs.

**Protein hit**	**Br homologs (s)**^**‡**^	**Organism**	**Score**	**P(U)**	**Sample**	**Accession**
BrPGIP1.1		*B. rapa* ssp. *pekinensis*	177	3(1)	8	MW264492
			172	3(1)	9	
			136	2(1)	12	
BrPGIP3		*B. rapa* ssp. *pekinensis*	193	2(2)	13	ACP28176
			130	1(1)	12	
			59	1(1)	10	
BrPGIP6		*B. rapa* ssp. *pekinensis*	106	1(1)	1	AAX68500
Polygalacturonase inhibitor protein 14	BrPGIP1, -1.1, -3 or -7	*B. napus*	155	2(1)	11	ABX46560.1
BrPGIP-like1		*B. rapa* ssp. *pekinensis*	394	6(5)	11	XP_009120892
			103	2(2)	12	
			73	1(1)	14	
Unnamed protein product	BrPGIP-like5	*B. rapa*	247	3(3)	11	VDC72802.1
DNA damage-repair/toleration protein DRT100		*B. rapa*	546	7(4)	12	XP_009145581.1
			521	7(3)	11	
			469	6(4)	8	
Hypothetical protein BRARA_H00290		*B. rapa*	59	1(1)	15	RID49491.1
			53	1(1)	13	
			53	1(1)	16	
Leucine-rich repeat extensin-like protein 4		*B. rapa*	61	1(1)	21	XP_033144188.1
Leucine-rich repeat receptor-like serine/threonine-protein kinase BAM1		*B. rapa*	406	6(5)	15	XP_009114790.1
			270	4(4)	14	
			145	2(2)	17	
Probable LRR receptor-like serine/threonine-protein kinase At1g56130		*B. rapa*	80	1(1)	15	XP_009105697.1
			70	1(1)	14	
			53	1(1)	13	

*Only protein hits from Brassicaceae were displayed. For each hit, the Mascot score (Score) as well as the number of matching peptides [P(U), number of unique peptides in the respective sample in brackets] is listed. If a hit was detected in more than one sample, those with the three highest Mascot scores were displayed. ^‡^Corresponding *B. rapa* ssp. *pekinensis* homologous genes were displayed for PGIPs and PGIP-like proteins (for protein alignments, see Supplementary Figure 6). For a full list of interacting proteins, see Supplementary Table 5.*

**TABLE 2:** Extracellular LRR protein hits from a pull-down assay of PCO_GH28-3 with *B. rapa* ssp. *pekinensis* CWPs.

**Protein hit**	**Br homolog**^**‡**^	**Organism**	**Score**	**P(U)**	**Sample**	**Accession**
BrPGIP-like1		*B. rapa* ssp. *pekinensis*	657	7(2)	11	XP_009120892
			616	8(5)	10	
			582	6(2)	12	
PREDICTED: polygalacturonase inhibitor 1-like	BrPGIP-like1	*Brassica oleracea* var. *oleracea*	488	1(1)	11	XP_013617122.1
			417	1(1)	12	
			210	1(1)	13	
BrPGIP-like5		*B. rapa* ssp. *pekinensis*	419	4(4)	10	XP_009146639
			359	6(4)	13	
			189	3(3)	14	
DNA damage-repair/toleration protein DRT100		*B. rapa*	540	7(3)	13	XP_009145581.1
			409	6(4)	12	
			125	2(1)	16	
Leucine-rich repeat receptor-like serine/threonine-protein kinase BAM1		*B. rapa*	309	5(4)	14	XP_009114790.1

*Only protein hits from Brassicaceae were displayed. For each hit, the Mascot score (Score) as well as the number of matching peptides [P(U), number of unique peptides in the respective sample in brackets] is listed. If a hit was detected in more than one sample, those with the three highest Mascot scores were displayed. ^‡^Corresponding *B. rapa* ssp. *pekinensis* homologous genes were displayed for PGIPs and PGIP-like proteins (for protein alignments, see Supplementary Figure 6). For a full list of interacting proteins, see Supplementary Table 6.*

BrPGIP3 bound to PCO_GH28-1, which correlates with our previous findings (Kirsch et al., 2012; Haeger et al., 2020). Furthermore, we detected BrPGIP1.1 and BrPGIP6 binding to and thus representing novel putative inhibitors of PCO_GH28-1 (see section “Materials and Methods” for distinction between BrPGIP1 and -1.1). Additionally, two PGIP-like proteins, BrPGIP-like1 and -like5, interacted with PCO_GH28-1 (Table 1). Interestingly, PCO_GH28-3 did not interact with any BrPGIP. Instead, we detected BrPGIP-like1 and -like5 binding to the pseudoenzyme (Table 2). For those PGIP and PGIP-like protein hits, where cultivar-dependent differences in the amino acid sequences affected the Mascot peptide matching, we identified the corresponding homologs in our *B. rapa* ssp. *pekinensis* cultivar (Tables 1, 2 and Supplementary Figure 6).

In conclusion, we not only confirmed the interaction of BrPGIP3 with PCO_GH28-1, but we also identified BrPGIP1.1 and -6 as potential inhibitors of this beetle PG. This is the first study showing that a PG pseudoenzyme was not targeted by PGIPs. Furthermore, we detected BrPGIP-like1 and -like5, two proteins remarkably similar to “classical” PGIPs, interacting with both PCO_GH28-1 and -3.

### Phylogeny of PGIPs and PGIP-like Proteins

Our pull-down assay revealed a number of LRR proteins interacting with beetle PG family members. Interestingly, these proteins resemble several characterized PGIPs (Supplementary Figure 2) and show either a high sequence similarity to known PGIPs from *B. napus* (Hegedus et al., 2008) and *B. rapa* ssp. *pekinensis* (Haeger et al., 2020) or to the recently described PGIP-like protein Bra-FLOR1 from *B. rapa* ssp. *pekinensis* (Liu et al., 2019). To get a better understanding of the evolutionary history of Brassicaceae PGIPs and PGIP-like proteins, we reconstructed the phylogenetic relationships between these sequences.

**FIGURE 2:**
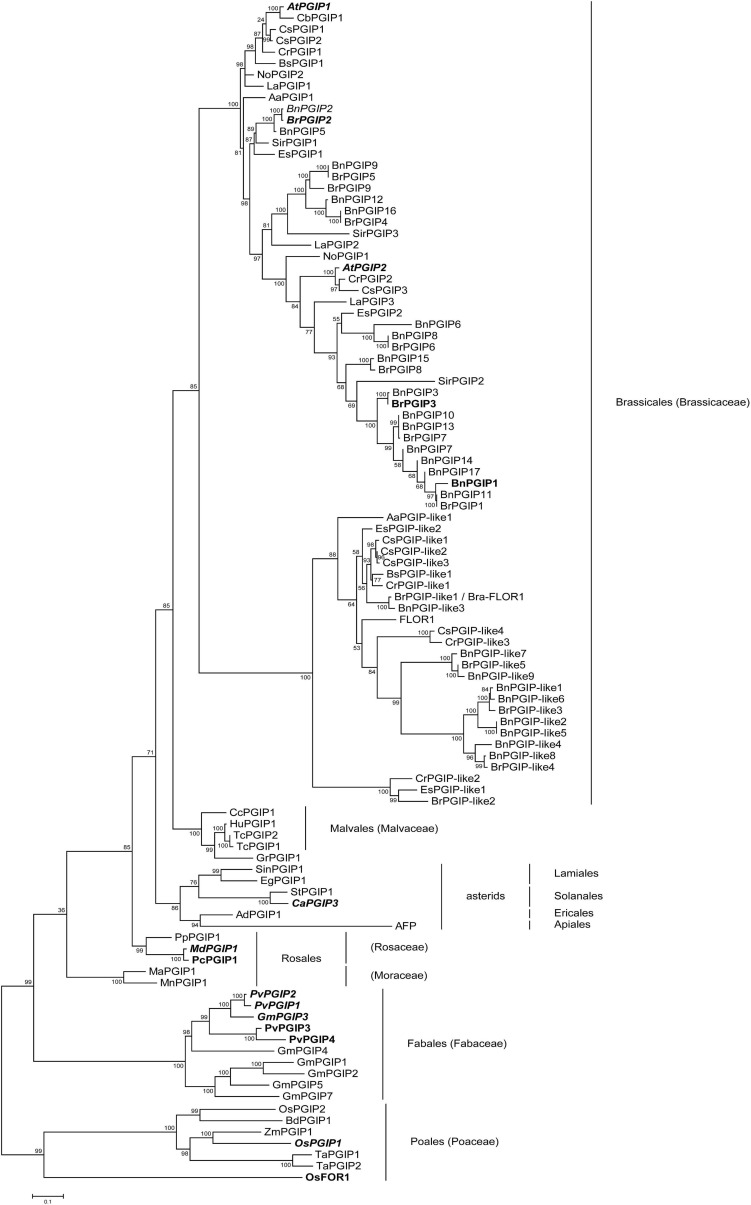
Phylogenetic relationships of various PGIPs and PGIP-like proteins. A full list of abbreviations and sequences used from GenBank and other sources can be found in Supplementary Table 3. PGIPs from Poales were used as an outgroup. Ultrabootstrap values are displayed for each node. Proteins printed in bold have been previously expressed in plants or heterologous systems and were tested positively for their inhibitory activity against microbial or insect PGs. Proteins with italicized names were overexpressed *in planta* and helped transgenic crops resist phytopathogenic fungi, oomycetes or bacteria.

Our phylogenetic tree contains 97 PGIPs and PGIP-like proteins from 29 species from eight different orders (Figure 2 and Supplementary Table 3). For an outgroup, we used seven PGIPs from three Poales species. In our phylogenetic analysis, all important nodes are well supported with ultrabootstrap values ≥ 85.

According to our phylogeny (Figure 2), PGIPs originated from a common ancestor and duplicated lineage-specifically. In the Brassicaceae, we see an early split into two well-supported clades: PGIPs and PGIP-like/FLOR1 proteins. PGIPs, which have previously been shown to inhibit a PG, cluster only in the “PGIP” clade. It is also apparent that most proteins of this clade have not yet been functionally characterized. The genes for PGIP-like/FLOR1 proteins duplicated and evolved independently from those in the “PGIP” clade. Only two proteins have been characterized in the “PGIP-like/FLOR1” clade. Both FLOR1 from *A. thaliana* (Torti et al., 2012) and Bra-FLOR1 from *B. rapa* ssp. *rapa* (Liu et al., 2019) – the latter is 100% identical to BrPGIP-like1 in our *B. rapa* ssp. *pekinensis* cultivar – have been associated with developmental processes. We found PGIP-like proteins in several Brassicaceae species from different genera (e.g., *B. napus*, *Camelina sativa*, and *Capsella rubella*), indicating that these genes are common throughout Brassicales plants.

Altogether, our study provides the most comprehensive phylogeny of PGIPs available to date and offers a broad overview of PGIPs of several species. In the Brassicaceae, compared to other plant lineages, we see not only an expansion of PGIPs but also an ancient separation into two clades: PGIPs and PGIP-like/FLOR1 proteins. Thus, our phylogeny together with the results from the pull-down assay raises the question of whether BrPGIP-like proteins retained PG-inhibiting activity or whether they evolved novel functions.

### Differential Regulation of BrPGIPs in Response to Mechanical Wounding and Beetle Feeding

**FIGURE 3:**
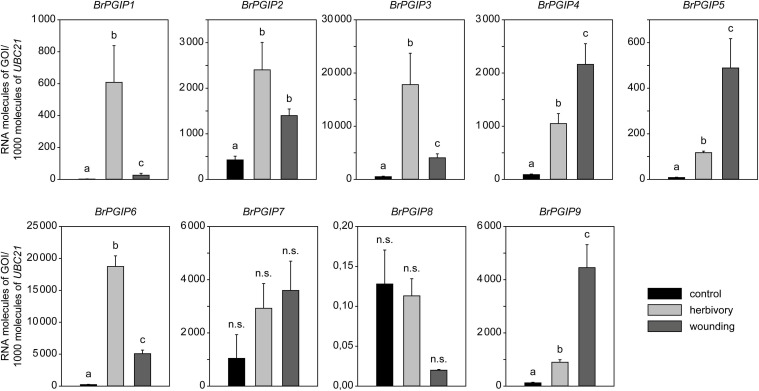
Regulation of *BrPGIPs* in response to *P. cochleariae* feeding and mechanical wounding. The expression levels of the *BrPGIPs* were quantified by RT-qPCR in untreated control plants (black), after *P. cochleariae* feeding (light gray) and mechanical wounding (dark gray). Transcript abundance is expressed as RNA molecules of the gene of interest (GOI) per 1000 RNA molecules of reference gene *UBIQUITIN-CONJUGATING ENZYME 21* (*UBC21*). Gene expression levels were compared between the three treatments (*n* = 5). Letters indicate significant differences (*p* < 0.05) between the groups based on an ANOVA on ranks or ANOVA followed by a Student–Newman–Keuls *post hoc* test. Error bars indicate the standard error of the mean (SEM). n.s., not significant.

Heterologous expression of the newly identified candidate proteins was challenging, and a comprehensive study of one-to-one interaction assays and functional characterization of these putative inhibitors of beetle PGs proved impossible. To nonetheless improve our understanding of whether these LRR proteins contribute to a defense reaction to herbivory, we analyzed the expression level of nine *BrPGIPs* in response to *P. cochleariae* feeding. Non-treated as well as mechanically wounded plants were used as controls (Figure 3).

In general, all *BrPGIPs* but *BrPGIP8* were expressed in *B. rapa* ssp. *pekinensis* leaves. In the untreated control plants, the *BrPGIPs* had different basal expression levels. *BrPGIP1 and -5* were barely expressed (<10 RNA molecules of GOI/1000 molecules of reference gene), whereas the other *BrPGIPs* showed a considerably higher constitutive expression (Supplementary Figure 7). In response to both *P. cochleariae* feeding and wounding, all *BrPGIPs*, except for *BrPGIP7*, were significantly upregulated. Remarkably, three different patterns of induction were observed. *BrPGIP2* was induced similarly by wounding and insect feeding. The expression of *BrPGIP4*, *-5*, and *-9* was higher in wounded plants compared to in those exposed to herbivore treatment (2-, 4-, and 5-fold, respectively). Conversely, *BrPGIP1*, *-3*, and *-6* were more strongly induced by *P. cochleariae* feeding than by wounding (23-, 4-, and 4-fold, respectively). Strikingly, in response to herbivory, *BrPGIP3* and *-6* showed the highest induction (35- and 80-fold higher than in the untreated control plants, respectively) (Figure 3). This pattern correlates well with the identification of these particular BrPGIPs in the pull-down assay with PCO_GH28-1 *in vitro*, further stressing their putative role as beetle PG inhibitors.

### BrPGIP-like1 Is Induced by Beetle Feeding and Interacts With a PG Pseudoenzyme

The function of BrPGIP-like proteins in *B. rapa* ssp. *pekinensis* is unknown. As we did for the *BrPGIPs*, we performed an expression analysis of *BrPGIP-like1* and *-like5* to assess their potential role in the context of beetle herbivory.

In untreated control plants, *BrPGIP-like1* and *-like5* showed low levels of basal expression, like most of the *BrPGIPss* (Supplementary Figure 7). *BrPGIP-like1* was induced in response to *P. cochleariae* feeding but not mechanical wounding, whereas *BrPGIP-like5* was unresponsive to both treatments.

**FIGURE 4:**
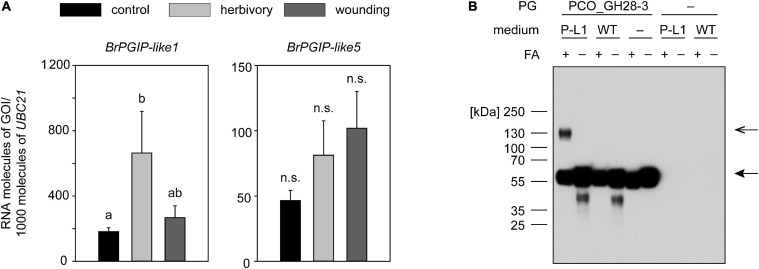
Regulation of *BrPGIP-like genes* in response to *P. cochleariae* feeding and mechanical wounding, and interaction of BrPGIP-like1 with PCO_GH28-3. **(A)** The expression levels of BrPgip-like1 and -like5 were quantified by RT-qPCR in untreated control plants (black), after *P. cochleariae* feeding (light gray) and mechanical wounding (dark gray), respectively. Transcript abundance is expressed as RNA molecules of interest (GOI) per 1000 RNA molecules of reference gene *UBIQUITIN-CONJUGATING ENZYME 21* (*UBC21*). Gene expression levels were compared between the three treatments (*n* = 5). Letters indicate significant differences (*p* < 0.05) between the groups based on an ANOVA on ranks or ANOVA followed by a Student–Newman–Keuls *post hoc* test. Error bars indicate the SEM. n.s., not significant. **(B)** Culture medium from BrPGIP-like1-expressing (P-L1) as well as wild-type (WT) yeast was incubated with PCO_GH28-3 and cross-linked with formaldehyde (FA). PCO_GH28-3 was detected in a western blot with an anti-V5 antibody. Arrows indicate the expected size of BrPGIP-like1 (closed arrowhead) and PCO_GH28-3–BrPGIP-like1 complex (open arrowhead).

In contrast to the upregulation of *BrPGIP1*, *-3*, and *-6* by beetle feeding, we cannot conclude that the induction of *BrPGIP-like1* by herbivory illustrated the difference between wounding and feeding treatments because the difference was not statistically significant; however, *BrPGIP-like1* was more induced by herbivory than wounding, showing a trend (Figure 4A).

Even though the heterologous expression of BrPGIP-like proteins turned out to be as challenging as the production of BrPGIPs, we were able to express recombinant BrPGIP-like1. As the protein was still highly unstable, we used it directly from the expression medium in an *in vitro* interaction study with PG family members from *P. cochleariae*. A band the size of the combined molecular weight of PCO_GH28-3 and BrPGIP-like1 was visible on a western blot when the proteins were cross-linked with formaldehyde (Figure 4B). No such band was detected when formaldehyde was omitted from the assay or in the control samples, such as medium from wild-type yeast and single protein incubations. This observation indicates a specific binding of BrPGIP-like1 to the pseudoenzyme PCO_GH28-3 and confirms the interaction of these proteins detected in the pull-down assay. No interaction of BrPGIP-like1 was detected with any of the other *P. cochleariae* PG family members, even, surprisingly, PCO_GH28-1 (Supplementary Figure 8).

Altogether, we confirmed that BrPGIP-like1 interacted with PCO_GH28-3 *in vitro* and demonstrated that *BrPGIP-like1* was induced in response to herbivory. Our findings indicate that cue-specific expression patterns as well as the ability to bind to PG family members may be conserved not only for members of the “PGIP” but also for members of the “PGIP-like/FLOR1” clade.

## Discussion

### BrPGIP1.1 and -6 Are Novel Putative Inhibitors of Beetle PGs

Using an unbiased pull-down assay with *B. rapa* ssp. *pekinensis* CWPs, we identified BrPGIP1.1, -3, and -6 as promising candidates for inhibitors of the beetle PG PCO_GH28-1. In our previous study, BrPGIP3 inhibited several beetle PGs, most efficiently PCO_GH28-1, as well as beetle gut PG activity (Haeger et al., 2020). Future heterologous expression and individual *in vitro* testing will reveal if our newly identified PGIP candidates possess inhibitory activities toward PGs as well.

Since heterologous expression of PGIPs was challenging, we performed a comprehensive analysis of gene regulation in response to various stresses to indicate putative functions of *B. rapa* ssp. *pekinensis* PGIPs. Unfortunately, due to the different sampling times, stresses and downstream quantification methods, comparing *PGIP* gene expression between studies is difficult. In general, the number of PGIP-encoding genes and their regulation varies drastically. In comparison to other plants, the large number of PGIPs and PGIP-like proteins in the genus Brassica is remarkable and most likely caused by polyploidization (Cheng et al., 2014). In *A. thaliana*, both *AtPGIPs* are upregulated in response to *P. cochleariae* feeding, wounding, phytopathogenic fungi and bacteria (De Lorenzo et al., 2001; Ferrari et al., 2003; Kirsch et al., 2020). Both AtPGIPs inhibit fungal PGs (Ferrari et al., 2003) and negatively influence weight gain in *P. cochleariae* larvae (Kirsch et al., 2020), thus appearing as “allrounders” in coping with various stresses (De Lorenzo and Ferrari, 2002). In *B. rapa* ssp. *pekinensis* and its close relative *B. napus*, however, some *PGIPs* are upregulated by certain cues while being unresponsive to others (Li et al., 2003; Hegedus et al., 2008; Hwang et al., 2010). Our qPCR data correlate with this pattern of a potential subfunctionalization and specialization among these PGIPs. *BrPGIP2* was induced to a similar extent by wounding and feeding. BrPGIP2 and its ortholog in *B*. *napus* BnPGIP2 have been shown to contribute to plant resistance to a bacterial and fungal phytopathogen, respectively (Hwang et al., 2010; Bashi et al., 2013) *BrPGIP4*, *-5*, and *-9* were induced more by wounding than herbivory. Thus, these BrPGIPs may play a role in a more general defense response or may be targeted against phytopathogens. In contrast, *BrPGIP1*, *-3*, and *-6* responded specifically to *P. cochleariae* feeding. This herbivory-induced upregulation matches perfectly with BrPGIP1.1, -3, and -6 binding to PCO_GH28-1 in the pull-down assay. In *B. napus*, *BnPGIP1* responded strongly to flea beetle (Coleoptera: Chrysomelidae) feeding (Li et al., 2003), and we previously characterized BrPGIP3 to be a versatile inhibitor of *P. cochleariae* PGs (Haeger et al., 2020). Altogether, these findings suggest that BrPGIP1.1, -3, and -6 are involved in plant defense against herbivorous beetles.

A well-characterized subfunctionalization of PGIPs against insects and fungi was studied in the bean *Phaseolus vulgaris* (D’Ovidio et al., 2004b; Frati et al., 2006). PvPGIP1 and -2 strongly inhibited several fungal PGs, whereas PvPGIP3 and -4 were less active against them. Instead, PvPGIP3 and -4, but not PvPGIP1 and -2, inhibited PG activity from multiple mirid bugs. Interestingly, PvPGIP3 and -4 did not achieve complete inhibition: their reduction of total mirid bug PG activity ranged between 10% and 42% (D’Ovidio et al., 2004b; Frati et al., 2006). Likewise, BrPGIP3 reduced the activity of *P. cochleariae* endo-PGs as well as gut PG activity by from 22 to 51% (Haeger et al., 2020). It would be interesting to investigate if a combination of BrPGIPs, particularly BrPGIP1.1, -3, and -6, reduces PG activity even more and what impact such inhibition would have on beetle fitness. On the other hand, the expanded set of PG family members in *P. cochleariae* could represent a strategy to evade an inhibition of PGs by plant PGIPs.

### BrPGIP-like Proteins – New Players in the Beetle Pectin Degradation Pathway

Catalytically inactive PG pseudoenzymes have been associated with the pectin degradation pathway in *P. cochleariae*. The downregulation of these pseudoenzymes by RNAi decreased the efficiency of food-to-energy conversion in larvae and prolonged the developmental period (Kirsch et al., 2019). The precise biological role of these pseudoenzymes and their ecological function, however, remains unknown. The pseudoenzyme PCO_GH28-3 did not interact with any BrPGIPs in our pull-down assay. Accordingly, BrPGIP3 did not bind to PCO_GH28-3 in a previous western blot-based one-to-one interaction assay (Haeger et al., 2020). This indicates that “classical” BrPGIPs might not target catalytically inactive PG family members. Although the structure of catalytically inactive PG family members remains to be resolved, we hypothesize that structural changes in pseudoenzymes compared to their active PG counterparts may be responsible for this absence of binding. Such structural alterations are further supported by the inability of pseudoenzymes – in contrast to active PGs – to bind to pectin substrates, which we demonstrated for PCO_GH28-3 and which has also been shown for pseudoenzymes from the rice weevil *Sitophilus oryzae* (Kirsch et al., 2016).

Surprisingly, we detected BrPGIP-like1 and -like5 binding to both PCO_GH28-3 and -1 in our pull-down assay. Given the number of peptides detected by LC-MS/MS, one may speculate that these BrPGIP-like proteins bound even more to PCO_GH28-3 than to -1. This is supported by our cross-linking assay with all *P. cochleariae* PG family members, which showed that BrPGIP-like1 could be seen interacting with PCO_GH28-3 but not -1. That we detected BrPGIP-like1 using mass spectrometry but not western blot can be explained by the methods’ sensitivities, which is substantially higher for mass spectrometry and allows detection of proteins below the detection limit of a western blot. On the other hand, the protein tag used for detection may be concealed due to the protein–protein interaction in the western blot.

BrPGIP-like proteins are closely related to “classical” PGIPs. The nominal distinction is mostly arbitrary, since most proteins have not been functionally characterized and, technically, can be termed “PGIP” only after a PG-inhibiting activity has been demonstrated. Nonetheless, many LRR proteins are called “PGIPs” merely based on sequence similarities. Previously published phylogenies of *PGIPs* are limited as these included only a small number of genes or were restricted to very few species (e.g., Li et al., 2003; D’Ovidio et al., 2004a, 2006; Hegedus et al., 2008; Hwang et al., 2010); only a few of these studies included PGIP-like proteins at all (e.g., De Lorenzo et al., 2001; Li et al., 2003; Ahsan et al., 2005; Janni et al., 2006). Our phylogenetic analysis revealed that PGIP-like proteins originated from a duplication event before Brassicaceae plants started to radiate. Both copies persisted as indicated by the two clades separating “classical” PGIPs and their “PGIP-like/FLOR1” counterparts, suggesting an important physiological function for both copies.

So far, only two Brassicales PGIP-like proteins have been characterized, one in *A. thaliana* (Torti et al., 2012) and a homolog in *B. rapa* ssp. *rapa* (Liu et al., 2019). In *A. thaliana*, FLOR1 promotes flowering (Torti et al., 2012), and, since its characterization, it has been the namesake for similar proteins. Interestingly, FLOR1 has been detected intracellularly and has interacted with a transcription factor (Gamboa et al., 2001; Acevedo et al., 2004) despite its secretion signal peptide. Bra-FLOR1, which is 100% identical with BrPGIP-like1 from *B. rapa* ssp. *pekinensis*, was recently linked to the formation of storage organs in *B. rapa* ssp. *rapa* (Liu et al., 2019). As we detected BrPGIP-like1 and -like5 in our CWP extracts and they, like Bra-FLOR1, were bioinformatically predicted to be extracellular proteins, we assume that these are localized within the cell wall. However, the localization of a PGIP is important for phytopathogens but probably not for chewing herbivores like *P. cochleariae*. Microorganisms secrete PGs into the apoplast, where they can be inhibited by extracellular PGIPs. In contrast, chewing insects masticate their food and intracellular as well as extracellular proteins encounter PGs in the digestive tract. Thus, it is not unlikely that such intracellular proteins have multiple functions, simultaneously serving as regulatory proteins and as PGIPs. In a similar way, lectins participate in various biological processes inside the plant cell but, released in response to herbivory, can have direct insecticidal effects or may inhibit larval growth and development (Vandenborre et al., 2011; Stahl et al., 2018). So far, no function for Bra-FLOR1 has been proposed in plants, which do not form storage hypocotyls, like *B. rapa* ssp. *chinensis* (Liu et al., 2019) or *B. rapa* ssp. *pekinensis*. In our study, BrPGIP-like1 and -like5 interacted with both tested PG family members, and *BrPGIP-like1* was induced by *P. cochleariae* feeding. Hence, we hypothesize that BrPGIP-like1/Bra-FLOR1 fulfills a dual function in both plant development and the pectin degradation pathway. As a BrPGIP-like protein has never before been linked to the pectin degradation pathway in herbivorous beetles, we can speculate that such proteins may also be interesting new candidates for PG inhibitors, not only for beetle- but also phytopathogen-derived PGs.

A PGIP with a dual function in both plant development and defense has been described for *Oryza sativa*. OsFOR1 inhibited a fungal PG from *Aspergillus niger* and was linked to floral organ development (Jang et al., 2003). In our phylogenetic analysis, OsFOR1 clusters together with other Poales-derived sequences. However, it represents the sister-clade to the other Poales PGIPs and shows approximately 40% sequence identity with the “classical” OsPGIP1 and -2, both of which have been shown to confer resistance to various phytopathogens (Janni et al., 2006; Chen et al., 2016; Wang et al., 2018). Whether Poales PGIP family members, like those of the Brassicaceae, cluster in two clades remains to be resolved. Nevertheless, a sub- or neofunctionalization of PGIPs does not seem exclusive to Brassicales plants. As described by Meyer et al. (1999), PGIPs, with their variable LRR domain, represent ideal candidates to adapt to novel ligands and functions. In that regard, AFP, an anti-freeze protein from carrot (*Daucus carota*), represents an extraordinary example of a PGIP-derived protein, one that has evolved to bind the non-proteinaceous ligand ice (Worrall et al., 1998; Meyer et al., 1999; Smallwood et al., 1999). Given the extended number of BrPGIPs and BrPGIP-like proteins, it is not surprising that some of these proteins may have evolved novel functions beyond targeting different PGs.

## Conclusion

In this study, we confirmed BrPGIP3 and identified BrPGIP1.1 and -6 as novel candidates of the beetle endo-PG PCO_GH28-1. In contrast, the PG pseudoenzyme PCO_GH28-3 was not targeted by any BrPGIP. Interestingly, we detected BrPGIP-like1 and -like5 binding to both PCO_GH28-1 and -3, and *BrPGIP-like1* being induced by beetle herbivory. Brassicaceous PGIP-like proteins separated before crucifer radiation and evolved independently from their “classical” PGIP counterparts; until now, these proteins have been associated with regulatory processes. Our results are the first to link these BrPGIP-like proteins to the pectin degradation pathway and to PG pseudoenzymes. This introduces a novel and fascinatingly versatile class of proteins to the complex, co-evolving interplay between PGs, PG pseudoenzymes and PGIPs.

## Data Availability Statement

The datasets presented in this study can be found in online repositories. The names of the repository/repositories and accession number(s) can be found in the article/Supplementary Material.

## Author Contributions

WH, YP, and RK conceived the study and designed the experiments. WH and RK performed the experiments. NW performed the LC-MS/MS. SG-J and YP performed the bioinformatic analyses. NS created the phylogeny. WH, YP, and RK analyzed the data. WH, YP, and RK wrote the manuscript with contributions from all co-authors. All authors read and approved the final manuscript.

## Conflict of Interest

The authors declare that the research was conducted in the absence of any commercial or financial relationships that could be construed as a potential conflict of interest.

## Footnotes

1 http://www.lrrfinder.com/lrrfinder.php

2 https://www.ebi.ac.uk/interpro/

3 https://blast.ncbi.nlm.nih.gov/Blast.cgi

4 http://brassicadb.org/brad/

5 http://iqtree.cibiv.univie.ac.at

## Supplementary Material

Supplementary Figure 1 Purification of PCO_GH28-1 and -3 and heat-dependent fragmentation of PCO_GH28-1.

Supplementary Figure 2 Alignment of PGIPs and PGIP-like proteins.

Supplementary Figure 3 Predicted functional classes from *B. rapa* ssp. *pekinensis* cell wall proteins (CWPs).

Supplementary Figure 4 Binding of *P. cochleariae* PG family members PCO_GH28-1 and -3 to pectic substrates.

Supplementary Figure 5 SDS-PAGE of pull-down assay of *B. rapa* ssp. *pekinensis* CWPs with an empty column (negative control).

Supplementary Figure 6 Alignment of BrPGIPs and BrPGIP-like proteins with homologs from different *B. rapa* ssp. *pekinensis* cultivars and Brassica species.

Supplementary Figure 7 Expression of *BrPGIPs* and *BrPGIP-like genes* in untreated *B. rapa* ssp. *pekinensis*.

Supplementary Figure 8 Interaction assay of PCO_GH28-1, -2, -4, -5, -6, -8, -9 with BrPGIP-like1-containing culture medium.

Supplementary Table 1 List of primers.

Supplementary Table 2 Fragmentation of PCO_GH28-1: nanoLC-MS/MS Mascot Results.

Supplementary Table 3 List of protein sequences used for phylogeny.

Supplementary Table 4 *Brassica rapa* ssp. *pekinensis* cell wall protein extraction: nanoLC-MS/MS Mascot Results.

Supplementary Table 5 Pull-down elution PCO_GH28-1: nanoLC-MS/MS Mascot Results.

Supplementary Table 6 Pull-down elution PCO_GH28-3: nanoLC-MS/MS Mascot Results.

Supplementary Table 7 Pull-down elution negative control: nanoLC-MS/MS Mascot Results.
