# Supplementary material for: New Players in the Interaction Between Beetle Polygalacturonases and Plant Polygalacturonase-Inhibiting Proteins: Insights From Proteomics and Gene Expression Analyses
Source: Front Plant Sci. 2021 Jun 4;12:660430. doi: 10.3389/fpls.2021.660430 (PMC8213348; doi:10.3389/fpls.2021.660430)
Supplement: Supplementary Figure 1 — Purification of PCO_GH28-1 and -3 and heat-dependent fragmentation of PCO_GH28-1. [file Data_Sheet_1.pdf]

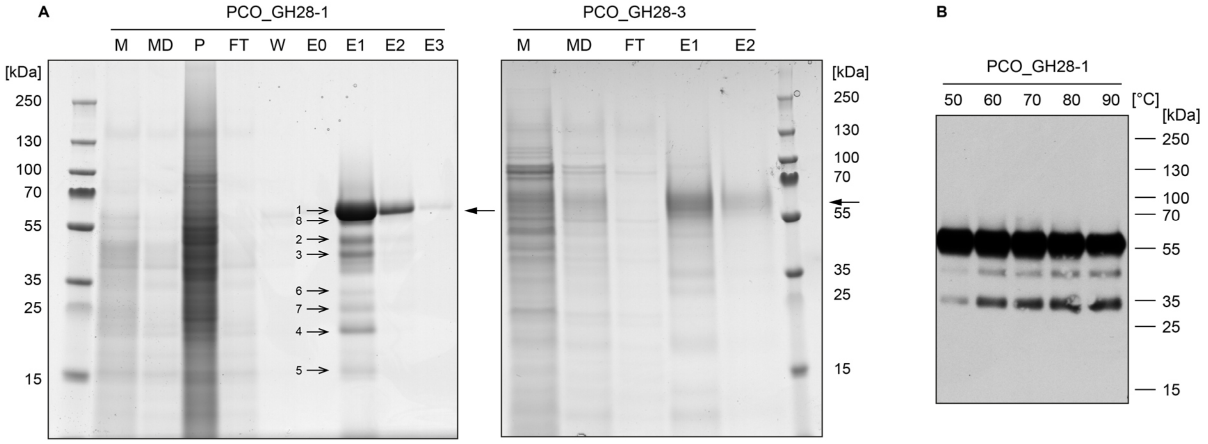

Supplementary Figure 1: Purification of PCO_GH28-1 and -3 and heat-dependent fragmentation of PCO_GH28-1. A) SDS-PAGE and Coomassie staining of exemplary metal ion affinity purification of PCO_GH28-1 and -3 via the His_6 tags. Their expected sizes are indicated with an arrow (closed arrowhead). Medium (M), dialyzed medium (MD), precipitated protein (P), flow-though (FT), wash (W) and elution fractions (E0 – E3). Additional bands in the purification of PCO_GH28-1 (arrows with open arrowheads, 1 – 8) were analyzed by MS/MS (see Supplementary Table 2) and identified as fragments of PCO_GH28-1. B) PCO_GH-28-1 was treated with increasing temperatures during sample preparation for SDS-PAGE and detected on the western blot with an anti-V5 antibody.

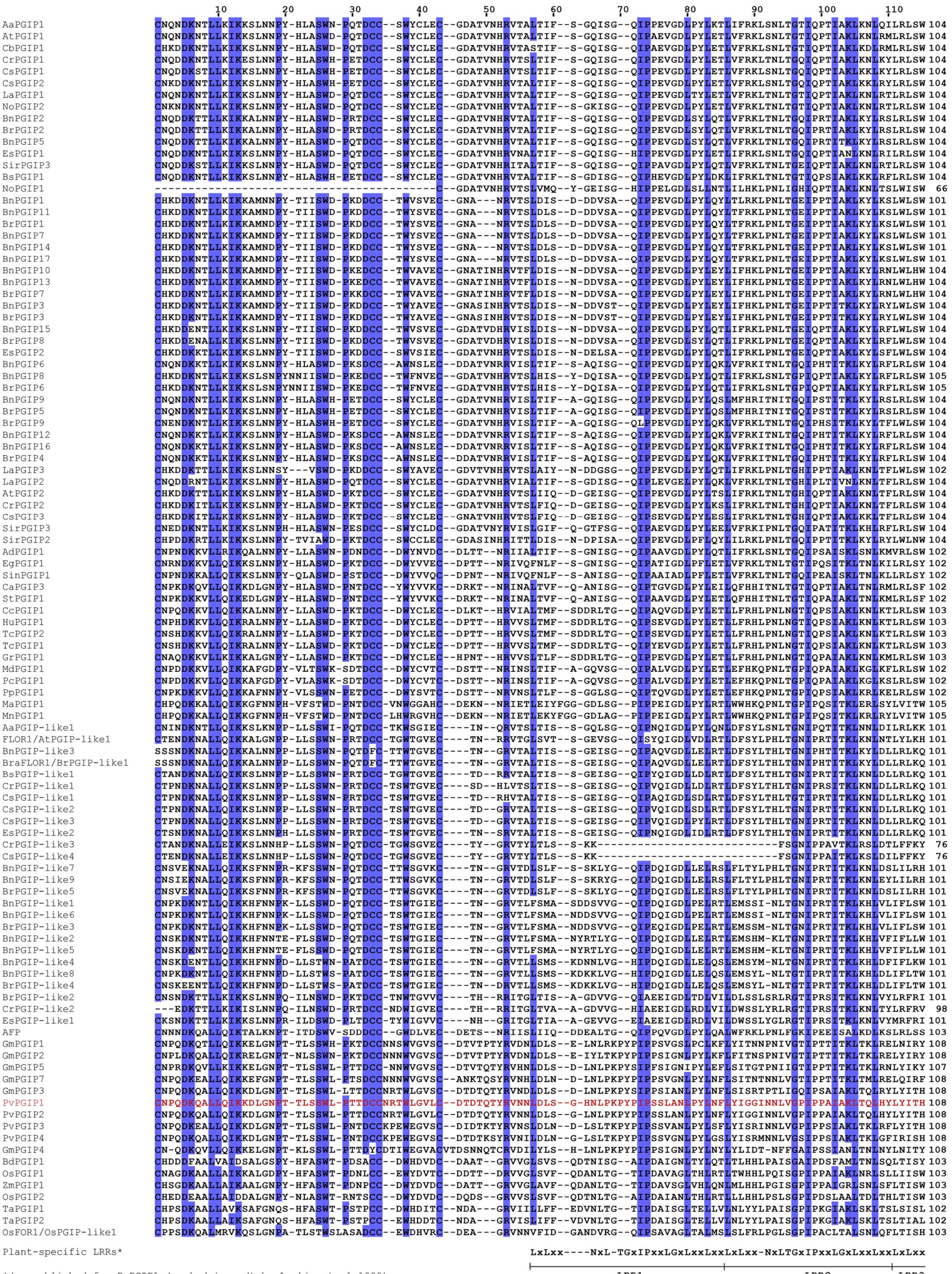

*(as published for PvPGIP1 (marked in red) by Leckie et al 1999)

```
                      120       130       140       150       160       170       180       190       200       210       220
AaPGIP1               TNLTGPVPDFISQLKNLQFLDLSYNDLSGSIPASLSLLPNLLSLDLSRNKLAGSIPETFG-LLPE-KV-----PDLY-LSHNQLSGSIPKS-LGNLDFNRIDFSRNKLEGDASML 210
AtPGIP1               TNLTGPIPDFISQLKNLEFLELSFNDLSGSIPSSLSTLPKILALELSRNKLTGSIPESFG-SFPG-TV-----PDLR-LSHNQLSGPIPKS-LGNIDFNRIDLSRNKLQGDASML 210
CbPGIP1               TNLTGPIPDFISQLKNLEFLELSFNDLSGSIPSSLSTLPKILALELSRNKLTGSIPESFG-SFPG-TV-----PDLR-LSRNQLSGPIPKS-LGNVDFNRIDLSRNELQGDASML 210
CrPGIP1               TNLTGPIPDFISQLKKLEFLDLSFNDLSGSIPSSLSTLPNIMALELSRNKLTGSIPESFG-SFPG-AV-----PDLR-LSHNQLSGPIPKS-LGNLDFNRIDLSRNKLEGDASML 210
CsPGIP1               TNLTGPIPDFISQLKNLEFLELSFNDLSGSIPSSLSTLPKIMALELSRNKLTGSIPESFG-SFPG-TV-----PDLR-LSHNQLSGPIPKS-LGNLDFNRIDLSRNKLEGDASML 210
CsPGIP2               TNLTGPIPDFISQLKNLEFLELSFNDLSGSIPSSLSTLPKIMALELSRNKLTGSIPESFG-SFPG-TV-----PDLR-LSHNQLSGPIPKS-LGNLDFNRIDLSRNKLEGDASMF 210
LaPGIP1               TNLTGPIPDFISQLKNLQFLDLSFNNLSGSIPSSLSLLPNILSLQLSRNKLTGSIPESFG-SFPV-KV-----PDLY-LSHNKLSGSIPNS-LGNLDFNRIDISRNKLEGDASML 210
NoPGIP2               TNLTGPIPDFISQLKNLQFLELSFNDLSGSIPSSLSMLPNILSLQLSRNKLTGSIPESFG-SFPG-QV-----PDLY-LSHNQLSGSIPKS-LGNLDFNRLDFSRNKLEGDASML 210
BnPGIP2               TNLTGPVPGFLSELKNLQWVDLSFNDLSGSIPSSLSLLPNLVSLDLSRNKLTGSIPESFG-SFPA-KV-----PDLY-LSHNQLSGYIPKT-LGNLDFSKIDFSRNKLGGDASML 210
BrPGIP2               TNLTGPVPGFLSELKNLQWVDLSFNDLSGSIPSSLSLLPNLVSLDLSRNKLTGSIPESFG-SFPA-KV-----PDLY-LSHNQLSGYIPKT-LGNLDFSKIDFSRNKLGGEASML 210
BnPGIP5               TNLTGPVPGFLSELKNLQFIDLSFNDLSGSVPSSLSLLPNLLSLDLSRNKLTGSIPESFG-SFPA-KV-----PDLY-LSHNQLSGYIPKT-LGNLDFNRIDFSRNKLGGDASML 210
EsPGIP1               TNLTGPIPGFLSQLKNLQFLDLSFNGLTGSIPSSLSLFPNLLSLDLSRNKLTGPIPESFG-SFQA-KV-----PDLY-LSHNELSGSIPKS-LGNLDFNRIDFSRNKLQGDASML 210
SirPGIP3              TNLTGPVPGFLSQLKNLQFLDLSFNDLSGSVPSSLSLLPNILSLDLSRNKLTGSIPKSFG-SFPA-NV-----PDLY-LSHNQLSGSIPKS-LGNLDFNRIDFSRNKLQGDASIL 210
BsPGIP1               TNLTGPIPDFLSQLKNLQFLDLSFNDLSGSIPSSLSLLPNIGALELSRNKLTGSIPESFG-SFPG-TV-----PDLR-LSHNQLSGSIPKS-LGNLDFNRIDFSRNKLEGDASML 210
NoPGIP1               TNLTGPVPEFLSQLKNLEYINLSFNNLTGSIPTSLASLHKLEFLELSRNKLTGPIPESFG-SFSG-KV-----PDLF-LSHNQLSGSIPKS-LGNLDFNRIDLSRNKLQGDASIL 172
BnPGIP1               NSLTGPVPEFLSQLKNLEYINLSFNKLSGSIPGSLSLLPKLDFLELSRNKLTGPIPESFG-SFKR-AA-----YGIY-LSHNQLSGPIPKS-LGNVDFNTIDLSRNKLEGDASML 207
BnPGIP11              NSLTGPVPEFLSQLKNLEYINLSFNKLSGSIPGSLSLLPKLDFLELSRNKLTGPIPESFG-SFKR-TV-----YGIY-LSHNQLSGPIPKS-LGNIDFNTIDLSRNKLEGDASML 207
BrPGIP1               NSLTGPVPEFLSQLKNLEYINLSFNKLSGSIPGSLSLLPKLDFLELSRNKLTGPIPESFG-SFKR-XV-----YGIY-LSHNQLSGPIPKS-LGNIDFNTIDLSRNKLEGDASML 207
BnPGIP7               NSLTGPVPEFLSQLKNLQYIDLSFNDLSGSIPGSLSLLPKLEILDLSRNKLTGSIPESFG-SFKG-VM-----YALF-LSHNQLSGSIPKS-LGNLDINQIDLSRNKLEGDASML 207
BnPGIP14              NSLTGPVPEFLSQLKNLEYINLSFNKLSGSIPGSLSLLPKLDFLELSRNKLTGPIPESFG-SFKR-TV-----YGIY-LSHNQLSGSIPKS-LGNLDINQIDLSRNKLEGDASML 207
BnPGIP17              NSLTGPVPEFLSQLKNLEYINLSFNKLSGSIPGSLSLLPKLDFLELSRNKLTGPIPESFG-SFKR-TV-----YGIY-LSHNQLSGPIPKS-LGNIDFNTIDLSRNKLEGDASML 207
BnPGIP10              NNLTGPVPEFLSQLKNLQYIDLSFNDLSGSIPGSLSLLPKLEILDLSRNKLTGSIPESFG-SFKG-VM-----YALF-LSHNQLSGSIPKS-LGNLDINQIDLSRNKLEGDASML 210
BnPGIP13              NNLTGPVPEFLSQLKNLQYIDLSFNDLSGSIPGSLSLLPKLEILDLSRNKLTGSIPESFG-SFKG-VM-----YALF-LSHNQLSGSIPKS-LGNLDINQIDLSRNKLEGDASML 210
BrPGIP7               NNLTGPVPEFLSQLKNLQYIDLSFNDLSGSIPGSLSLLPKLEILDLSRNKLTGSIPESFG-SFKG-VM-----YALF-LSHNQLSGSIPKS-LGNLDINQIDLSRNKLEGDASML 210
BnPGIP3               NNLSGPVPEFLSQLKNLEYINLSFNKLSGSIPGSLSLLPKLEFLELSRNKLTGSIPESFG-SFKG-VV-----YALY-LSHNQLSGSIPKS-LGNLDINQIDLSRNKLEGDASML 210
BrPGIP3               NNLSGPVPEFLSQLKNLEYINLSFNKLSGSIPGSLSLLPKLEFLELSRNKLTGSIPESFG-SFKG-VV-----YALY-LSHNQLSGSIPKS-LGNLDINQIDLSRNKLEGDASML 210
BnPGIP15              THLTGPVPEFLSQLKDLEYINLSFNDLSGSIPGSLSLLPKLGILELSRNKLTGSIPESFG-AFKG-VV----PPELF-LSHNQLSGSIPKS-LGNLDFHRIDFSRNKLEGDASMM 211
BrPGIP8               TNLTGPVPEFLSQLKDLEYINLSFNDLSGSIPGSLSLLPKLGILELSRNKLTGSIPESFG-AFKG-VV----PPEIF-LSHNQLSGSIPKS-LGNLDFHRIDFSHNKLEGDASMM 211
EsPGIP2               TNLTGPVPAFLSQLKNLEYINLSFNDLSGSIPSSLSLLPKLGFLELSRNKLTGPIPESFG-SFQG-QV-----PDLF-LSHNQLSGSIPKS-LGKLDIYRIDLSRNKLQGDASML 210
BnPGIP6               TNLTGPVPEFLSQLMDLAYLNLSFNYFSGSIPSSLSLLPKLEYLELSRNKLTGPIPELFR-SFPG-KA-----PDLF-LSHNQLNGSIPKS-LGKLDFYRIDLSHNKLKGDASML 210
BnPGIP8               TNLTGPIPDFFSQLKNLQYIDLSYNDLSGSIPTSLALLPKLEYLELSRNKLTGPIPELFR-SFPG-KA-----PDLF-LSHNQLNGSIPKS-LGKLDFYRIDLSHNKLKGDASML 211
BrPGIP6               TNLTGPIPDFFSQLKNLQYIDLSYNDLSGSIPTSLALLPKLEYLELSRNKLTGPIPELFR-SFPG-KA-----PDLF-LSHNQLNGSIPKS-LGKLDFYRIDLSHNKLKGDASML 211
BnPGIP9               LNLTGPVPEFLSQLMNLEYLSLSFNQLSGSIPSSLALLPKLSYVDLSRNKLTGTIPESFG-SFPA-EL-----AYLI-LSHNQLSGSIPKS-LGNLDFNRIDFSRNKFTGDASML 210
BrPGIP5               LNLTGPVPEFLSQLMNLEYLSLSFNQLSGSIPSSLALLPKLSYVDLSRNKLTGTIPESFG-SFPA-EL-----AYLI-LSHNQLSGSIPKS-LGNLDFNRIDFSRNKFTGDASML 210
BrPGIP9               TNLTGPVPDFLSQLVNLDYLNLSFNELSGSVPSSLSLLPKLTYIDLSRNKLTGTIPESFG-SFPG-EV-----PYLI-LSHNQLSGSIPKS-LGNLDFNRIDFSRNKLTGDASML 210
BnPGIP12              TNLTGPVPEFLSQLMDLAYLNLSFNYFSGSIPSSLSLLPKLSYVDLSRNKLTGTIPESFG-SFSG-EV-----PDLF-LSHNQLSGSIPKS-LGNLDFNRIDFSRNRFIGDASML 210
BnPGIP16              TNLTGPVPEFLSQLMDLAYLNLSFNYFSGSIPSSLSLLPKLSYVDLSRNKLTGTIPESFG-SFSG-EV-----PDLF-LSHNQLSGSIPKS-LGNLDFNRIDFSRNRFIGDASML 210
BrPGIP4               TNLTGPVPEFLSQLMDLAYLNLSFNYFSGSIPSSLSLLPKLSYVDLSRNKLTGTIPESFG-SFSG-EV-----PDLF-LSHNQLSGSIPKS-LGNLDFNRIDFSRNRFIGDASML 210
LaPGIP3               TNLTGPVPEFLSQLKNLEYINLSFNKLSGPIPSSLSGSLHKLGFLELSRNKLTGPIPESFG-SFTG-EV-----PDLF-LSHNQLSGPIPKS-LGNLDFNRIDLSRNKLEGDASIL 208
LaPGIP2               TNLSGPVPEFLSQLKNLEYMDLSFNDLSGSIPSSLSSSLPKLGFLDLSRNKLTGPIPESFG-SFLG-ED-----PYLL-LSHNQLSGSIPKS-LGNLDFNKIDLSRNKLQGDASML 210
AtPGIP2               TNLTGPVPEFLSQLKNLEYIDLSFNDLSGSIPSSLSSLRKLEYLELSRNKLTGPIPESFG-TFSG-KV-----PSLF-LSHNQLSGTIPKS-LGNPDFYRIDLSRNKLQGDASIL 210
CrPGIP2               TNLTGPIPEFLSQLKNLEYIDLSFNDLSGYIPSSLSSLPKLEYLELSRNKLTGPIPESFG-SFSG-KV-----PSLF-LSHNQLSGTIPKS-LGNSDFYRIDLSRNKLQGDASIL 210
CsPGIP3               TNLTGPVPEFLTQLKNLEYIDLSFNDLSGSIPSSLSSLRKLEYLELSRNKLTGPIPESFG-SFSG-KV-----PSLF-LSHNQLSGTIPKS-LGNSDFYRIDLSRNKLQGDASIL 210
SirPGIP3              TNLTGPVPGFLSQLMNLAYLDLSFNQLSGSIPSSLSLLPKLNYIDLSRNKLTGTIPESFG-SFPS-TG-----PYLF-LSHNQLTGSIPRS-LGNLDFYKIDFSRNKLQGDASML 210
SirPGIP2              NNLTGPVPEFLSELKNLEYINLSFNNLSGSIPSSLSLLPKLEYVDLGRNKLTGSIPESFG-SFK--VA-----PYLL-LSHNQLTGSIPKS-LGNLNYPKIDLSRNKLEGDASVL 209
AdPGIP1               TNLSGPVPSFFSQLKNLTFLDLSFNDLTGSIPSSLSKLTNLDAIHLDRNKLTGPIPNSFG-EFTG-QV-----PDLY-LSHNQLTGPIPKT-LGDLNFTVIDVSRNMLSGDISFM 208
EgPGIP1               TNLSGAIPSFLSQLENLTFLDLSFNNLTGEIPPSLSLLKNLDALHLDRNKLTGKIPDSIG-KFAG-KT-----PDLY-FSHNQLTGAVPRS-LGDLNFTVLDFSRNKLEGDASFL 208
SinPGIP1              TNLSGPIPTFLSQLKNLTFLDLSFNNLTGSIPPSISNLPNLDALHLDRNKLTGSIPDSLG-KFTE-KV-----PDLY-LSHNHLTGSVPRS-LGDLNFTVLDFSRNQLEGDISFL 208
CaPGIP3               TNLTGPIPEFLSQLKKLTLLELDYNQLTGTIPASLSQLPNLNAIYLDRNKLTGQIPESFG-KFKG-QI-----PDIY-LSHNSLTGQVPRS-LGDLNFSTLDFSRNKLEGDVSFL 208
StPGIP1               TNLSGPIPEFLSQLKNLTLLELNYNQLTGTIPSSLSQLPNLLAIYLDRNKLTGSIPESFG-RFKGPNI-----PDLY-LSHNSLTGKVPTS-LGDLNFSRLDFSRNKLEGDVSFL 209
CcPGIP1               TNLSGPVPDFLSQLKNLTFLDLSFNNLSGSIPSSLSKLPNLLALHLDRNKLTGSIPESLG-RFKG-NV-----PDLY-LSHNKLTGTIPAS-LGNLDFNTIDFSRNLLEGDASML 209
HuPGIP1               TNLSGTVPDFLSQLKNLTYLDLSFNNLSGNIPSSLSSLPNLEALHLDRNKFTGSIPESFG-MFQG-KA-----PDLF-LSHNKLTGTIPVS-LGNMDFNTIDLSRNMLEGDASML 209
TcPGIP2               TNLSGTVPDFLSQLKNLTYLDLSFNNLSGTIPSSLSSLPNLEALHLDRNKFTGSIPESLG-MFQG-KA-----PDLF-LSHNKLTGTIPVS-LGNMDFNTIDLSRNMLEGDASML 209
TcPGIP1               TNLSGAVPDFLSQLKNLTYLDLSFNNLSGTIPSSLSSLPNLEALHLDRNKFTGSIPESLG-MFQG-KA-----PDLF-LSHNKLTGTIPVS-LGNMDFNTIDLSRNMLEGDASML 209
GrPGIP1               TNLSGPVPNFLSQLKNLTYLDLSFNNLSGSIPSSLSTLPNLEDLHLDRNKLTGTIPESFG-MFPS-KK-----LYLFI-LSHNKLSGTIPAS-LANMDFNTIDLSRNLLEGDPSVL 210
MdPGIP1               TNLSGSVPDFLSQLKNLTFLDLSFNNLTGAIPSSLSQLPNLNALHLDRNKLTGHIPKSLG-QFIG-NV-----PDLY-LSHNQLSGNIPTS-FAQMDFTSIDLSRNKLEGDASVI 208
PcPGIP1               TNLSGSVPDFLSQLKNLTFLDLSFNNLTGAIPSSLSELPNLGALRLDRNKLTGHIPISFG-QFIG-NV-----PDLY-LSHNQLSGNIPTS-FAQMDFTSIDLSRNKLEGDASVI 208
PpPGIP1               TNISGSVPDFLSQLKNLTFLELSFNNLTGSIPSSLSQLPNLVALHLDRNKLTGHIPKSFG-EFHG-SV-----PDLY-LSHNQLSGTIPTS-LAKLDFSTVDFSRNKLEGDASMI 208
MaPGIP1               ANLSGPIPDFLSEIKSLVSIEFSFNNLTGSIPSSLSRLPNLTGLALDRNKLTGPIPDSFG-EFQG-FS-----FYLK-LSHNQLSGKIPAS-LGKKDFTYVDLSRNKLEGDASVL 211
MnPGIP1               ANLSGPIPDFLSEIKSLVSIEFSFNNLTGQIPSSLSRLPNLTGLVLDRNKLTGPIPDSFG-EFQG-SH-----FYLK-LSHNQLSWKIPAS-LGNKDFNYVDLSWNKLEGGAAVL 211
AaPGIP-like1          TKLSGSIPGFISQLKNVTFLDLSFNKLTGLIPGSLSQMPKLEAIQINDNKLTGSIPASFG-SFVG-KV-----PNLY-LFNNQLSGKVPES-LSKYDFNALDLSGNNLIGDGSMF 207
FLOR1/AtPGIP-like1    TSLSGPIPDYISELKSLTFLDLSFNQFTGPIPGSLSQMPKLEAIQINDNKLTGSIPNSFG-SFVG-NV-----PNLY-LSNNKLSGKIPES-LSKYDFNAVDLSGNGFEGDAFMF 207
BnPGIP-like3          TNLSGSIPDFISELKSVTFLDLSFNQFTGPIPGSLSQMPKLGALQINDNKLTGSIPNSFG-SFVG-TV-----PNLY-LFNNKLSGKVPES-LSKYDFNAVDLSGNSFIGDGSMF 207
BraFLOR1/BrPGIP-like1 TNLSGYIPDFISELKSVTFLDLSFNQFTGPIPGSLSQMPKLDAIQINDNKLTGSIPNSFG-SFVG-NV-----PNLY-LFNNKLSGKVPES-LSKYDFNAVDLSGNSFTGDGSMF 207
BsPGIP-like1          TNLSGPIPDYISELKSVTFLDLSFNQFTGPIPGSLSQLPKIQAIQINDNKLTGSIPNSFG-SFVG-NV-----PNLY-LFNNKLSGKVPES-LSKYDFNAVDLSGNGFIGDGSMF 207
CrPGIP-like1          TNLSGSIPDYISELKTLTFLDLSFNQFTGPIPGSLSQMPKLQAIQINDNKLTGSIPNSFG-SFVG-NV-----PNLY-LFNNKLSGRVPES-LSKYDFNAVDLSGNGFMGDGSMF 207
CsPGIP-like1          TNLSGPIPDYISELKTVTFLDLSFNQFTGPIPGSLSQMPKLQAIQVNDNKLTGSIPNSFG-SFVG-NV-----PNLY-LFNNKLSGKVPES-LSKYDFNAVDLSGNGFIGDGSMF 207
CsPGIP-like2          TNLSGPIPDYISELKTVTFLDLSFNQFTGPIPGSLSQMPKLQAIQVNDNKLTGSIPNSFG-SFVG-NV-----PNLY-LFNNKLSGKVPES-LSKYDFNAVDLSGNGFIGDGSMF 207
CsPGIP-like3          TNLSGPIPDYISELKTVTFLDLSFNQFTGPIPGSLSQMPKLQAIQVNDNKLTGSIPNSFG-SFVG-NV-----PNLY-LFNNKLSGKVPES-LSKYDFNAVDLSGNGFIGDGSMF 207
EsPGIP-like2          NNLSGPIPEYISELKNVTFLDLSFNQFTGPIPGSFSQMPKLQAIQINDNKLTGSVPNSFG-SFVG-NV-----PNLY-LFNNKLSGKIPES-LSKYDFNAVDLSGNGFIGDGSMF 207
CrPGIP-like3          SNFSGPIPDNISDLKNLTYLGLSENQLTGPIPGSLSQMPKLAIHVNDNKLTGSIPNSFG-SFVG-NV-----PGLE-LSNNRLSGKIPES-LSKHDFNVVFLSGNSFVGDGSMF 182
CsPGIP-like4          SNFSGPIPDNISDLKNLTYLGLSENQLTGPIPGTLSQMPKLEAIHVNDNKLTGSIPNSFG-SFVG-NV-----PGLD-LSNNRLSGKIPES-LSKHDFNSVFLSGNSFVGDGSMF 182
BnPGIP-like7          NNLSGPIPDNIGELKKLSFLDLSYNQFTGPIPGSLSQMPMLETIKIDHNKLTGSVPNSFG-SFVG-NV-----PNLY-LSNNKLSGKIPSS-LSKNDFNGVFLSKNSFTGDGSMF 207
BnPGIP-like9          NNLSGLIPDNIGELKRLRFLDLSYNQFTGPIPGSLSQMPMLETIIVEHNKLTGSIPNSFG-SFVG-EV-----PNLY-LSNNKLSGKIPES-LSKNDFNGVFLSKNSFTGDGSMF 207
BrPGIP-like5          NNLSGPIPDNIGELKRLRFLDLSYNQFTGPIPGSLSQMPMLETIIVEHNKLTGSIPNSFG-SFVG-NV-----PNLY-LSNNKLSGNIPES-LSKNDFNGVFLSKNSFTGDGSMF 207
BnPGIP-like1          NKLSGPIPDYISELKSVTFLDISFNKFTGPIPGWLTQMPNLQTFQADNNSLTGPIPNSFG-SFVG-NV-----PNLF-LPYNKLSGKIPES-LSKIDFQAIVLKGNGFTGDGSMF 207
BnPGIP-like6          NRLSGPIPDYISELKSVTFLDISFNKFTGPIPGWLTQMPKLQTFQANNNSLTGPIPNSFG-SFVG-NA-----PNLF-LPYNKLSGKIPES-LSKIDFQAVVLKGNGFTGDGSMF 207
BrPGIP-like3          NKLSGPIPDYVSELKSVTFLDISFNKFTGPIPGWLTQMPKLHTFQADNNSLTGPIPNSFG-SFVG-NV-----PNVF-LPYNKLSGKIPES-LSKIDFQAIVLKGNGFTGDGSMF 207
BnPGIP-like2          NKLSGPIPDYISELKSVTFLDISFNKFTGPIPGWLTQMPKLQTFQADNNSLTGPVPNSFG-SFVG-SV-----PNLF-LKNNKLSGKIPES-LSKMDFQAVVLKGNGFTGDGSMF 207
BnPGIP-like5          NKLSGPIPDYISELKSVTFLDISFNKFTGPIPGWLTQMPKLQTFQADNNSLTGPVPNSFG-SFVG-SV-----PNLF-LKNNKLSGKIPES-LSKMDFQAVVLKGNGFTGDGSMF 207
BnPGIP-like4          NRLSGPIPEYISELKNVTFMDLSYNKFTGPVPGWLTQMPKLQTFQIDNNSLTGPIPNSFG-SFAD-SV-----PNLF-LKNNKLSGKIPES-LSKIDFQAVVLAGNSFTGDGSMF 207
BnPGIP-like8          NRLSGPIPEYISELKSVTFLDISFNKFTGPIPGWLTQMPNLQTFQADNNSLTGSIPNSFG-SFVG-NV-----PNLF-LPYNKLSGKIPES-LSKIDFQAVVLTGNSFTGDGSMF 207
BrPGIP-like4          NRLSGPIPEYISELKSVTFLDISFNKFTGPIPGWLTQMPNLQTFQADNNSLTGPIPNSFG-SFVG-NV-----PNLF-LPYNKLSGKIPES-LSKIDFQAVVLTGNSFTGDGSMF 207
BrPGIP-like2          TELSGPVPDYISQLQNVTFLDLSFNRFNGSIPGSISQMHRLETIQLSHNKLTGSIPESFG-SFAG-KI-----PKLY-LGNNHLSGEIPKS-LSKTNFNTVSLSGNNFSGDASMF 207
CrPGIP-like2          TELSGPIPEYISELQNVTFLDLSFNRFNGSIPGSISLMPKLEAVQLNRNKLTGSIPESFG-SFVG-KV-----PDLY-LGNNRLSGKIPKS-LSKSDFNTLSLSGNKLSGDASMF 204
EsPGIP-like1          TELSGPIPEYISELQNVTFLDLSFNRFNGSIPGSISQMPKLEAIQLNHNKLTGSIPESFG-SFLG-KV-----PDLF-LGNNHLSGKIPKT-LSKSDFNTLSLSGNNFSGDASMF 207
AFP                   TSLSGPVPLFFPQLTKLTCLDLSFNKLLGVIPPQLSTLPNLKALHLERNELTGEIPDIFG-NFAG-S------PDIY-LSHNQLTGFVPKT-FARADPIRLDFSGNRLEGDISFL 208
GmPGIP1               TNISGQIPHFLSQIKALGFLDLSNNKLSGNLPSWLPSLPDLYGISFDNNYISGPIPDLFA-SVSE-RF-----GFIS-LSGNRLIGKIPAS-LGKPDMKIVDLSRNMLEGDASVL 214
GmPGIP2               TSVSGQIPHFLSQMKTLQFLEFSNCKLSGNLPTWLPSLPDLYGVAFDNNRISGAIPDSFG-FVSK-RF-----GYMT-LSGNRLSGKIPSS-LGKLDLKTVDLSRNKLEGDASVL 214
GmPGIP5               TNVSGQIPRFLSQIKTLEFLDLSYNKLSGNLPAWLPSLPNLVGISFDGNRISGAIPDSFG-YFPK-SF-----VMLS-LSRNRLTGKIPAT-LAKLDVKFVYLSKNMLEGDASLL 213
GmPGIP7               TNVSGEIPHFLSQIKTLESIIFHYNNFSGNLPPWLPSLPNLYRVSLDGNRISGTIPDSFG-SFSD-SL-----KLMT-FSDNRLTGEIPAT-LAKLDFDFVDLSQNMLEGDASVL 214
GmPGIP3               TNVSGPIPDFLSQIKTLVTLDFSYNTLSGKLPASLASLPNLVGITFDGNQISGAIPDSYG-SFSK-LF-----TSMT-ISRNRLTGKIPAT-LAKLNLAFVDLSRNMLEGDASVL 214
PvPGIP1               TNVSGAIPDFLSQIKTLVTLDFSYNALSGTLPPSISSLPNLGGITFDGNRISGAIPDSYG-SFSK-LF-----TAMT-ISRNRLTGKIPPT-FANLNLAFVDLSRNMLEGDASVL 214
PvPGIP2               TNVSGAIPDFLSQIKTLVTLDFSYNALSGTLPPSISSLPNLVGITFDGNRISGAIPDSYG-SFSK-LF-----TSMT-ISRNRLTGKIPPT-FANLNLAFVDLSRNMLEGDASVL 214
PvPGIP3               TNVSGQIPNFLSQMKTLITIDFSYNALSGTLPPSISSLPNLLGISLDGNRISGTIPGSFG-SFPK-HF-----TVLT-LSRNRLTGNIPAT-LAKLELAFVDLSENMLEGDASVL 214
PvPGIP4               TNVSGQIPNFLSQMKSLITIDFSYNALSGTLPPSISSLPNLVGITLDGNRISGTIPGSFG-SFPK-HF-----TVLT-LSRNRLTGNIPTT-LAKLGLSFVDLSENMLEGDASVL 214
GmPGIP4               TNVSGTIPDFLSHIKTLVSIDFSYNNLSGNLPASLSSLPNLGEMIFTGNRISGAIPDSFG-SFSE-EL-----ILMR-LSRNRLTGKIPAT-LAKLNLRFLDLSRNMLEGDASVL 214
BdPGIP1               TGLTGPIPSFLSVLTELTLLDLSYNSLTGVIPASLADLPNLSSLNLRRNRLTGPIPLLLL-SNSP-DQ-----ASLL-LSHNNLSGTIPSE-FSTVNFTHIDISHNALTGDASCL 209
OsPGIP1               TAVSGPVPSFLGGLKSLTLLDLSFNSLTGAIPPSLAALPFLSGIDISRNRLTGPLPPALF-SKLN-TTQQG-GAYLR-LSRNNLTGGIPAE-YGGVAFE---------------- 197
ZmPGIP1               TGVSGPVPSFLGALTRLNQLDLSFNALTGAVPASLAALPSLYSIDISRNRLTGSLPPLLF-SRLD-KAQQ--EAYLR-LSHNNLTGSVPPE-FSAVAFAQVDLSRNAFAGDASGL 212
OsPGIP2               TAVSGPVPSFLANLTSLTMLDLSFNSLTGLIPPSLAALTNLSAIDISRNRLSGPIPPGLF-SNLQ-LQDDDDEVYLR-LSHNNLSGSVPADLLAANALAVDLSRNALTGDASAV 215
TaPGIP1               TSVSGPIPSFLGALTKLNDLTLSSNSLTGTIPASLAGLRYLDTIDLRRNRLTGTIPPLFYNSKSP-II-----PYLL-LSNNHLSGSIPTE-FAAVGFQFIDLSHNALTGDASVF 209
TaPGIP2               TAVSGPIPSFLGELTRLKELTLFSNSLSGTIPASLGGLRYLDSIELRRNRLTGTIPPLF--SKST-LL-----PVLL-LSNNHLSGSIPAE-FAAVGFLQVDLSHNALTGDASVF 207
OsFOR1/OsPGIP-like1   TNVSGVIPDSLARIRSLDSVDLSHNSLTGPIPNSFSDLPNLRSLDLRSNKLTGCIPA--G-LVQG-QF-----RSLI-LSYNQLTGPIPRD-DAQDEINTVDLSHNRLTGDASFL 207

Plant-specific LRRs   NxLTGxIPxxLGxLxxLxxLxLxxNxLTGxIPxxLGxLxxLxxLxLxxNxLTGxIPxxLG-xLxx-Lx-----x-Lx-LxxNxLTGxIPxx-LGxLxxLxLxLxxNxLTGxIPxx

                      |      LRR3      |        LRR4        |             LRR5              |          LRR6         |    LRR7    |
```

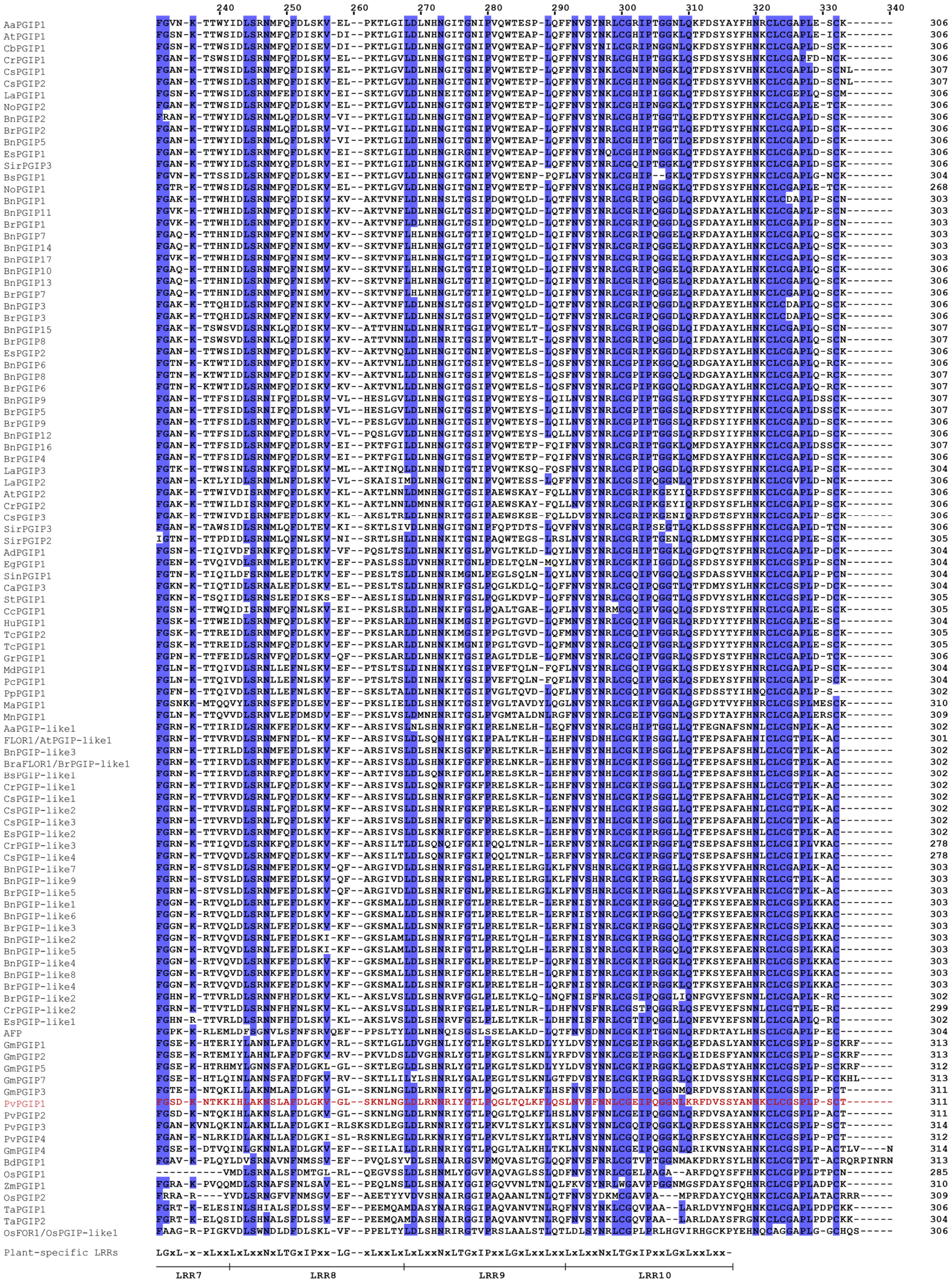

Supplementary Figure 2: Alignment of PGIPs and PGIP-like proteins. Conserved amino acid residues above 80% identity are highlighted in blue. Leucine-rich repeats (LRRs) were displayed below as reported by Leckie *et al.* (1999) for PvPGIP2 from *Phaseolus vulgaris* (highlighted in red). For full sequences, see Supplementary Table 3.

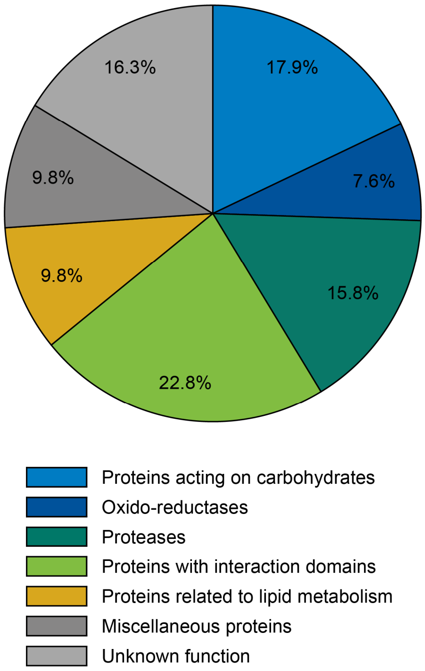

Supplementary Figure 3: Predicted functional classes from *B. rapa* ssp. *pekinensis* cell wall proteins (CWPs). The annotation of proteins is based on the presence of functional domains predicted by InterPro, GO Term and BLAST bioinformatics analysis. Analysis includes only proteins that possess a signal peptide for the secretory pathway according to SignalP and were predicted to be localized extracellularly by DeepLoc analysis. For a full list of identified proteins, see Supplementary Table 4.

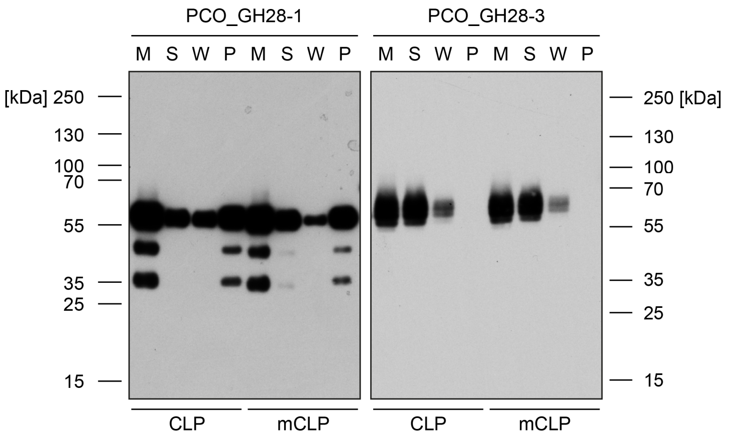

Supplementary Figure 4: Binding of *P. cochleariae* PG family members PCO_GH28-1 and -3 to pectic substrates. Culture medium containing PCO_GH28-1 and -3 (M) was incubated with cross-linked pectin (CLP) and methylated CLP (mCLP), respectively. Centrifugation separated the pectin pellet and associated proteins (P) from the supernatant (S). Unbound proteins were removed from the pellet by washing (W). The volumes applied to the western blot were chosen in such a way that the cumulative signals of S, W and P correspond to the initially used amount of M. The PG family members were detected in a western blot with an anti-V5 antibody. The additional bands below the PCO_GH28-1 signal in the western blot were analyzed by LC-MS/MS and assigned to fragments of the PG, which occur as an artefact of the sample boiling for SDS-PAGE (Supplementary Figure 1, Supplementary Table 2).

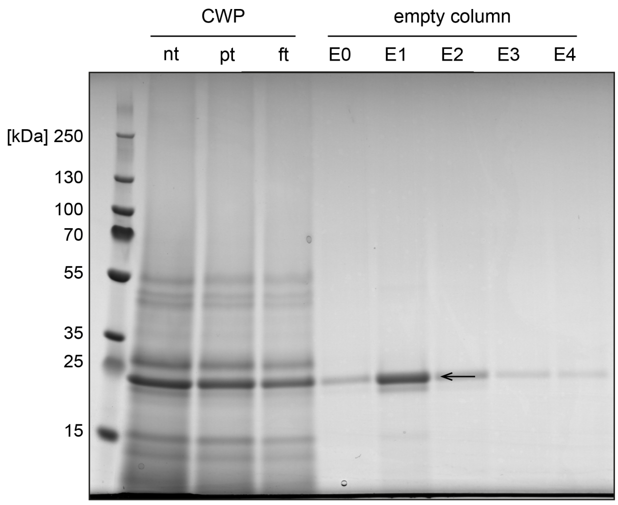

Supplementary Figure 5: SDS-PAGE of pull-down assay of *B. rapa* ssp. *pekinensis* CWPs with an empty column (negative control). Non-treated (nt) CWP extracts were pre-treated (pt) with column resin to reduce unspecific binding to the column and then passed over columns without immobilized protein. Proteins not interacting with the column material were in the flow-through (ft), while interacting proteins were eluted from the column analogous to the PG family member columns. Elution fraction E1 was analyzed by LC-MS/MS. For detailed information on interacting LRR and total proteins, see Supplementary Table 6. E0 – 4: elution fractions 1 – 4.

A

```
                                                10        20        30        40        50        60
BrPGIP1_BRA_Chifu-401-42                        MG-KTTILLLLLFALLLTTSLSKDLCHKDDKNTLLKIKKAMNDPYTIISWDPKDDCCTWY
BrPGIP_PGIP1.1_BRA_Cantonner_Witkrop            MG-KTTILLLLLFALLLTTSLSKDLCHKDDKNTLLKIKKAMNDPY---SWDPKDDCCTWY
BrPGIP3_BRA_Chifu-401-42                        MEGKTTTLLLLLFALLLTTSLSKDLCHKDDKNTLLKIKKAMNDPYTIISWDPKDDCCTWY
BrPGIP7_BRA_Chifu-401-42                        MD-KTTTLLLLLFALLLTTSLSKDLCHKDDKNTLLKIKKAMNDPYTIISWDPKKDCCTWV
BnPGIP14_BNA                                    MG-KTTILLLLLFALLLTTSLSKDLCHKDDKNTLLKIKKAMNDPYTIISWDPKDDCCTWY

                                                70        80        90        100       110       120
BrPGIP1_BRA_Chifu-401-42                        SVECGNA---NRVTSLDLSDDDVSAQIPPEVGDLPYLQYLTFRKLPNLTGEIPPTIAKLK
BrPGIP_PGIP1.1_BRA_Cantonner_Witkrop            SVECGNA---NRVTSLDLSDDDVSAQIPPEVGDLPYLQYLTFRKLPNLTGEIPPTIAKLK
BrPGIP3_BRA_Chifu-401-42                        AVECGNASINHRVTSLDISNDDVSTQIPPEVGDLPYLEYLIFHKLPNLTGEIPPTITKLK
BrPGIP7_BRA_Chifu-401-42                        AVECGNATINHRVTFLDISNDDVSAQIPPEVGDLPYLEYLIFHKLPNLTGEIPPTIAKLK
BnPGIP14_BNA                                    SVECGNA---NRVTSLDLSDDDVSAQIPPEVGDLPYLQYLTFRKLPNLTGEIPPTIAKLK

                                                130       140       150       160       170       180
BrPGIP1_BRA_Chifu-401-42                        YLKSLWLSWNSLTGPVPEFLSQLKNLEYINLSFNKLSGSIPGSLSLLPKLDFLELSRNKL
BrPGIP_PGIP1.1_BRA_Cantonner_Witkrop            YLKSLWLSWNSLTGPVPEFLSQLKNLEYINLSFNKLSGSIPGSLSLLPKLDFLELSRNKL
BrPGIP3_BRA_Chifu-401-42                        YLRYLWLSWNNLSGPVPEFLSQLKNLEYINLSFNKLSGSIPGSLSLLPKLEFLELSRNKL
BrPGIP7_BRA_Chifu-401-42                        YLRNLWLHWNNLTGPVPEFLSQLKNLQYIDLSFNDLSGSIPGSLSLLPKLEILDLSRNKL
BnPGIP14_14_BNA                                 YLKSLWLSWNSLTGPVPEFLSQLKNLEYINLSFNKLSGSIPGSLSLLPKLDFLELSRNKL

                                                190       200       210       220       230       240
BrPGIP1_BRA_Chifu-401-42                        TGPIPESFGSFKRTVYGIYLSHNQLSGSIPKSLGNIDFNTIDLSRNKLEGDASMLFGVKK
BrPGIP_PGIP1.1_BRA_Cantonner_Witkrop            TGPIPESFGSFKRAVYGIYLSHNQLSGSIPKSLGNIDFNTIDLSRNKLEGDASMLFGTKK
BrPGIP3_BRA_Chifu-401-42                        TGSIPESFGSFKGVVYALYLSHNQLSGSIPKSLGNLDINQIDLSRNKLEGDASMLFGAKK
BrPGIP7_BRA_Chifu-401-42                        TGSIPESFGSFKGVMYALFLSHNQLSGSIPKSLGNLDINQIDLSRNKLEGDASMLFGAQK
BnPGIP14_BNA                                    TGPIPESFGSFKRTVYGIYLSHNQLSGSIPKSLGNLDINQIDLSRNKLEGDASMLFGAQK

                                                250       260       270       280       290       300
BrPGIP1_BRA_Chifu-401-42                        TTWHIDLSRNMFQFDISKVKVAKTVNFLDLNHNGLTGSIPDQWTQLDLQTFNVSYNRLCG
BrPGIP_PGIP1.1_BRA_Cantonner_Witkrop            TTWHIDLSRNMFQFDISKVKVAKTVNFLDLNHNSLTGSIPDQWTQLDLQTFNVSYNRLCG
BrPGIP3_BRA_Chifu-401-42                        TTQHIDLSRNMFQFNISKVKVAKTVNFLDLNHNSLTGSIPVQWTQLDLQTFNVSYNRLCG
BrPGIP7_BRA_Chifu-401-42                        TTHNIDLSRNMFQFNISMVKVSKTVNFLHLNHNGLTGTIPIQWTQLDLQIFNVSYNRLCG
BnPGIP14_BNA                                    TTHNIDLSRNMFQFNISMVKVSKTVNFLHLNHNGLTGTIPIQWTQLDLQIFNVSYNRLCG

                                                310       320       330       340
BrPGIP1_BRA_Chifu-401-42                        RIPQGGDLQSFDAYAYLHNKCLCGAPLPSCNVKIQATDLYLNLPSE
BrPGIP_PGIP1.1_BRA_Cantonner_Witkrop            RIPQGGDLQIFDAYAYLHNKCLCGAPLPSCNVKIQATDLYLNLPSE
BrPGIP3_BRA_Chifu-401-42                        RIPQGGDLQRFDAYAYLHNKCLCDAPLQSCK
BrPGIP7_BRA_Chifu-401-42                        RIPQGGELQRFDAYAYLHNKCLCGAPLQSCK
BnPGIP14_BNA                                    RIPQGGELQRFDAYAYLHNKCLCGAPLQSCK
```

B

```
                                            10        20        30        40        50        60
BrPGIP-like1/Bra-FLOR1_BRA_Chifu-401-42   MKLLLHLSIFFTILFISLPSSHSSSSNDKNALLQIKKALNNPPLLSSWNPQTDFCTTWTG
BrPGIP_like1_BRA_Cantonner_Witkrop        MKLLLHLSIFFTILFISLPSSHSSSSNDKNALLQIKKALNNPPLLSSWNPQTDFCTTWTG
PREDICTED:PG_inhibitor_1-like_BOL         MTLLLHLSIFFTILFISLPSSHSSSSNDKNALLQIKKALNNPPLLSSWNPQTNFCTTWTG

                                            70        80        90       100       110       120
BrPGIP-like1/Bra-FLOR1_BRA_Chifu-401-42   VECTNGRVTALTISSGEISGQIPAQVGDLLELRTLDFSYLTHLTGNIPHTITKLKYLDLL
BrPGIP_like1_BRA_Cantonner_Witkrop        VECTNGRVTALTISSGEISGQIPAQVGDLLELRTLDFSYLTHLTGNIPHTITKLKYLDLL
PREDICTED:PG_inhibitor_1-like_BOL         VECTNGRVTALTISSGEISGQIPAQVGDLLELRTLDFSYLTHLTGNIPHTITKLKYLDLL

                                           130       140       150       160       170       180
BrPGIP-like1/Bra-FLOR1_BRA_Chifu-401-42   RLKQTNLSGYIPDFISELKSVTFLDLSFNQFTGPIPGSLSQMPKLDAIQINDNKLTGSIP
BrPGIP_like1_BRA_Cantonner_Witkrop        RLKQTNLSGYIPDFISELKSVTFLDLSFNQFTGPIPGSLSQMPKLDAIQINDNKLTGSIP
PREDICTED:PG_inhibitor_1-like_BOL         RLKQTNLSGYIPDFISELKSVTFLDLSFNQFTGPIPGSLSQMPKLGALQINDNKLTGSIP

                                           190       200       210       220       230       240
BrPGIP-like1/Bra-FLOR1_BRA_Chifu-401-42   NSFGSFVGKVPNLYLFNNKLSGKVPESLSKYDFNAVDLSGNSFTGDGSMFFGRNKTTIRV
BrPGIP_like1_BRA_Cantonner_Witkrop        NSFGSFVGKVPNLYLFNNKLSGKVPESLSKYDFNAVDLSGNSFTGDGSMFFGRNKTTIRV
PREDICTED:PG_inhibitor_1-like_BOL         NSFGSFVGIVPNLYLFNNKLSGKVPESLSKYDFNAVDLSGNSFIGDGSVFFGRNKTTIRV

                                           250       260       270       280       290       300
BrPGIP-like1/Bra-FLOR1_BRA_Chifu-401-42   DLSRNMFEFDLSKVKFARSIVSLDLSHNKIFGKFPRELNKLRLEHFNVSYNHLCGKIPSG
BrPGIP_like1_BRA_Cantonner_Witkrop        DLSRNMFEFDLSKVKFARSIVSLDLSHNKIFGKFPRELNKLRLEHFNVSYNHLCGKIPSG
PREDICTED:PG_inhibitor_1-like_BOL         DLSRNMFEFDLSKVKFARSIVSLDLSHNKISGKFPRELTKLRLEHFNVSYNHLCGKIPSG

                                           310       320
BrPGIP-like1/Bra-FLOR1_BRA_Chifu-401-42   GLLQTFEPSAFSHNLCLCGTPLKAC
BrPGIP_like1_BRA_Cantonner_Witkrop        GLLQTFEPSAFSHNLCLCGTPLKAC
PREDICTED:PG_inhibitor_1-like_BOL         GLLQTFEPSAFSHNLCLCGTPLKAC
```

C

```
                                             10         20         30         40         50         60
BrPGIP-like5/FLOR1_BRA_Chifu-401-42     MKLLLHLSIFFAILFISLPSSYSCNSVEKNALLQIKKSFNNPRKFSSWNPQTDCCTTWSG
BrPGIP_like5_BRA_Cantonner_Witkrop      MKLLLHLSIFFAILFISLPSSYSCNSVEKNALLQIKKSFNNPRKFSSWNPQTDCCTTWSG
unnamed_protein_product_BRA_Z1          MKLLLHLSIFFAILFISLPSSYSCNSVEKNALLQIKKSFNNPRKFSSWNPQTDCCTTWSG

                                             70         80         90        100        110        120
BrPGIP-like5/FLOR1_BRA_Chifu-401-42     VKCTNGRVTDLSLFSSKLYGQIPDQIGDLLELRSLFLTYLPHLTGNIPRTITKLKNLDSL
BrPGIP_like5_BRA_Cantonner_Witkrop      VKCTNGRVTDLSLFSSKLYGQIPDQIGDLLELRSLFLTYLPHLTGNIPRTITKLKNLEYL
unnamed_protein_product_BRA_Z1          VKCTNGRVTDLSLFSSKLYGQIPDQIGDLLELRSLFLTYLPHLTGNIPRTITKLKNLEYL

                                            130        140        150        160        170        180
BrPGIP-like5/FLOR1_BRA_Chifu-401-42     ILRHNNLSGPIPDNIGELKKLRFLDLSYNQFTGPIPGSLSQMPMLETIIVEHNKLTGSIP
BrPGIP_like5_Cantonner_Witkrop          ILRHNNLSGLIPDNIGELKKLRFLDLSYNQFTGPIPGSLSQMPMLETIIVEHNKLTGSIP
unnamed_protein_product_BRA_Z1          ILRHNNLSGPIPDNIGELKKLRFLDLSYNQFTGPIPGSLSQMPMLETIIVEHNKLTGSIP

                                            190        200        210        220        230        240
BrPGIP-like5/FLOR1_BRA_Chifu-401-42     NSFGSFVGEVPNLYLSNNKLSGNIPESLSKNDFNGVFLSKNSFTGDGSMFFGRNKSTVSL
BrPGIP_like5_BRA_Cantonner_Witkrop      NSFGSFVGEVPNLYLSNNKLSGNIPESLSKNDFNGVFLSKNSFTGDGSMFFGRNKSTISL
unnamed_protein_product_BRA_Z1          NSFGSFVGEVPNLYLSNNKLSGNIPESLSKNDFNGVFLSKNSFTGDGSMFFGRNKSTVSL

                                            250        260        270        280        290        300
BrPGIP-like5/FLOR1_BRA_Chifu-401-42     DLSRNMFEFDLSKVQFARGIVDLDLSHNRIFGNLPRELIELRGLKLFNVSHNRLCGKIPR
BrPGIP_like5_BRA_Cantonner_Witkrop      DLSRNMFEFDLSKVQFARGIVDLDLSHNRIFGNLPRELIELRGLKLFNVSHNRLCGKIPR
unnamed_protein_product_BRA_Z1          DLSRNMFEFDLSKVQFARGIVDLDLSHNRIFGNLPRELIELRGLKLFNVSHNRLCGKIPR

                                            310        320
BrPGIP-like5/FLOR1_BRA_Chifu-401-42     GGLLQSFKSYVFAHNRCLCGTPLKAC
BrPGIP_like5_BRA_Cantonner_Witkrop      GGLLQSFKSYVFAHNRCLCGTPLKAC
unnamed_protein_product_BRA_Z1          GGLLQSFKSYVFAHNRCLCGTPLKAC
```

Supplementary Figure 6: Alignment of BrPGIPs and BrPGIP-like proteins with homologs from different *B. rapa* ssp. *pekinensis* cultivars and Brassica species. CWPs for the pull-down assay were obtained from the *B. rapa* ssp. *pekinensis* (BRA) cultivar "Cantonner Witkrop," while sequences for mass spectrometry and bioinformatic analyses were obtained from the NCBI database and originate from the "Chifu-401-42" cultivar. If the Mascot analyses resulted in hits other than from the "Chifu-401-42" sequences, we aligned those together with the sequences from "Cantonner Witkrop." Matching peptides from the Mascot analyses were marked in blue, amino acid differences were highlighted in black. A) For the hit BnPGIP14 (ABX46560.1) from *B. napus* (BNA), one peptide matched to BrPGIP1 and -1.1, while the second matched BrPGIP3 and -7. B) One amino acid difference between the detected peptide and the BrPGIP-like1 sequences resulted in the identification of the homologous "Predicted: polygalacturonase inhibitor-like1" (XP_013617122.1) from *B. oleraceae* var. *oleracea* (BOL). C) All peptides match perfectly with the hit "unnamed protein product" (VDC72802.1) from the *B. rapa* cultivar "Z1" and BrPGIP-like5 from "Cantonner Witkrop," but differ in one amino acid from the BrPGIP-like5/FLOR1 sequence (XP_009146639) from "Chifu-401-42." Altogether, we are confident that discrepancies resulted from cultivar-dependent or even individual-dependent amino acid differences and that the proteins we identified were BrPGIP1.1 and -3 as well as BrPGIP-like1 and -like5.

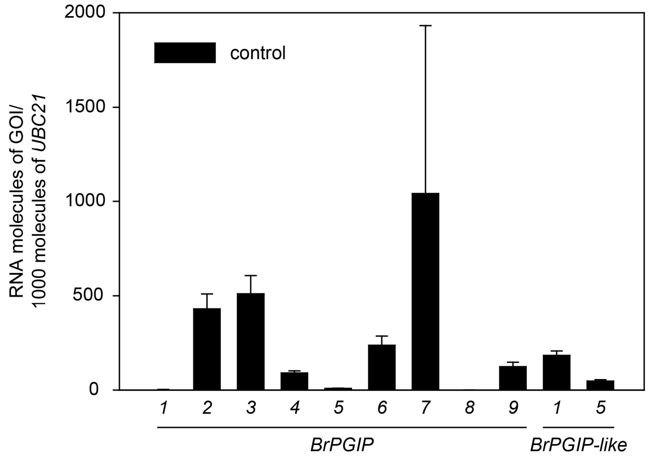

Supplementary Figure 7: Expression of *BrPGIPs* and *BrPGIP-like proteins* in untreated *B. rapa* ssp. *pekinensis*. The expression levels of the *BrPGIPs* and *BrPGIP-like proteins* were quantified by RT-qPCR, and transcript abundance was expressed as RNA molecules of the gene of interest (GOI) per 1000 RNA molecules of the reference gene *UBIQUITIN-CONJUGATING ENZYME 21 (UBC21)*. Error bars indicate the standard error of the mean (SEM).

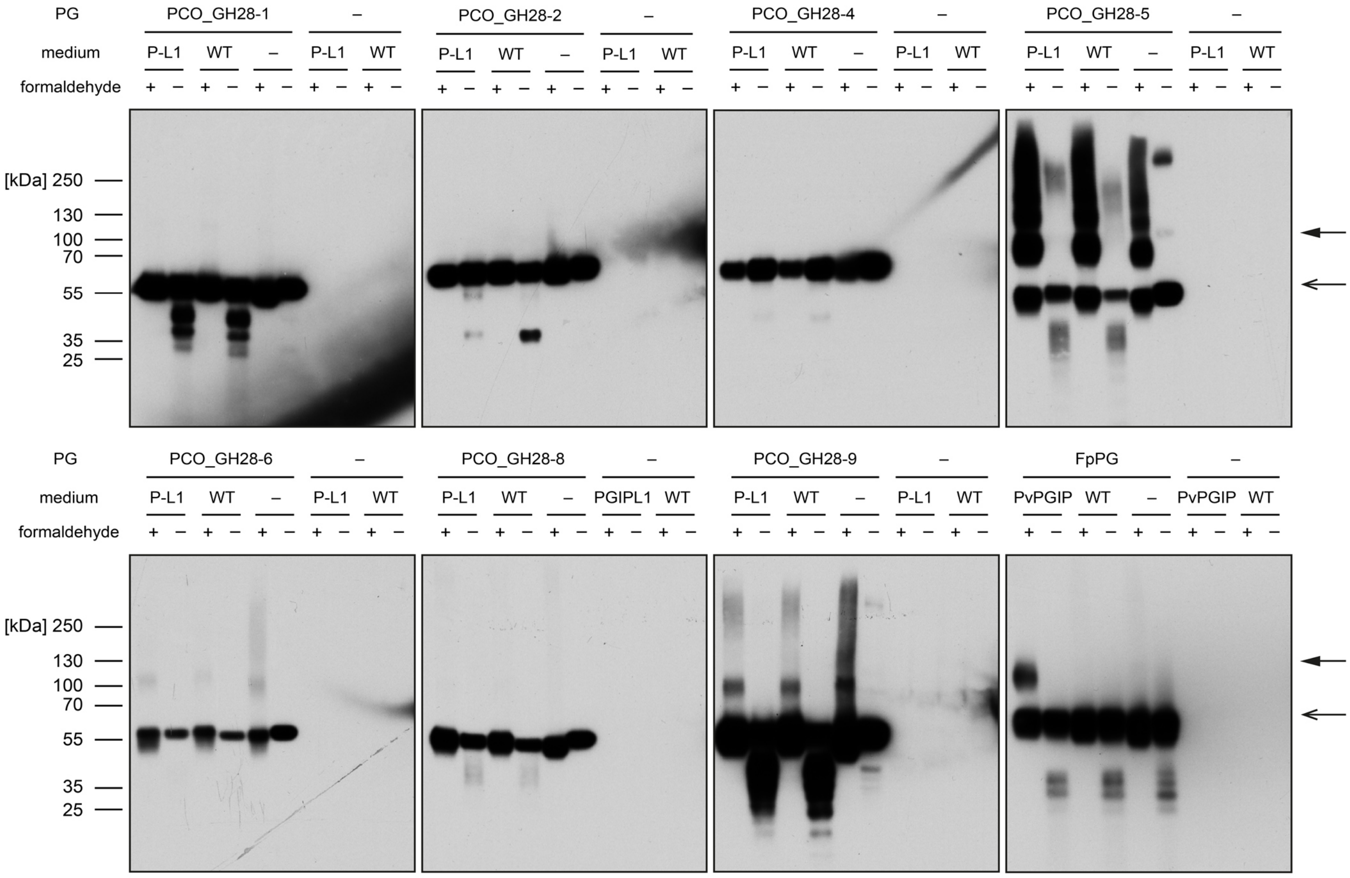

Supplementary Figure 8: Interaction assay of PCO_GH28-1, -2, -4, -5, -6, -8, -9 with BrPGIP-like1-containing culture medium. Culture medium from BrPGIP-like1-expressing (P-L1) as well as wild-type (WT) yeast was incubated with various *P. cochleariae* PG family members and cross-linked with formaldehyde. The PG family members were detected in a western blot with an anti-V5 antibody. Arrows indicate the expected size of PG family members (open arrowhead) and PG-PGIP complex (closed arrowhead).
